# Stepwise shifts underlie evolutionary trends in morphological complexity of the mammalian vertebral column

**DOI:** 10.1038/s41467-019-13026-3

**Published:** 2019-11-07

**Authors:** Katrina E. Jones, Kenneth D. Angielczyk, Stephanie E. Pierce

**Affiliations:** 1 Museum of Comparative Zoology and Department of Organismic and Evolutionary Biology, Harvard University, 26 Oxford Street, Cambridge, MA 02138 USA; 2 Integrative Research Center, Field Museum of Natural History, 1400 South Lake Shore Drive, Chicago, IL 60605-2496 USA

**Keywords:** Evolutionary theory, Palaeontology

## Abstract

A fundamental concept in evolutionary biology is that life tends to become more complex through geologic time, but empirical examples of this phenomenon are controversial. One debate is whether increasing complexity is the result of random variations, or if there are evolutionary processes which actively drive its acquisition, and if these processes act uniformly across clades. The mammalian vertebral column provides an opportunity to test these hypotheses because it is composed of serially-repeating vertebrae for which complexity can be readily measured. Here we test seven competing hypotheses for the evolution of vertebral complexity by incorporating fossil data from the mammal stem lineage into evolutionary models. Based on these data, we reject Brownian motion (a random walk) and uniform increasing trends in favor of stepwise shifts for explaining increasing complexity. We hypothesize that increased aerobic capacity in non-mammalian cynodonts may have provided impetus for increasing vertebral complexity in mammals.

## Abstract (web summary)

The mammalian vertebral column has become more complex over evolutionary time. Here, Jones and colleagues use phylogenetic modelling to show that this complexity increased in stepwise shifts likely driven by adaptations for increased aerobic capacity.

## Introduction

Understanding the remarkable complexity of living organisms is one of the most fundamental and alluring topics in biology. When examining the diversity of life through geologic time, from single-celled organisms to modern ecosystems, it seems clear that biological complexity has increased^1–3^. The tendency for increasing complexity through time is a widely held tenant of evolutionary biology and is often thought of as a ubiquitous long-term trend. However, it need not be the result of an actively-driven evolutionary process^4–6^. Instead, random variation combined with a fixed lower bound on complexity (the simplest organisms) could suffice to produce an overall increase through time passively via Brownian motion. Alternatively, increases due to shifts in ecology, phylogenetic composition, or developmental patterning may mimic long-term trends when examined over coarsely-resolved evolutionary timescales. Despite the extensive philosophical and theoretical discussion, few empirical tests of complexity increase in real biological systems exist^2,4^ (but see refs ^7,8^). Consequently, the ubiquity of long-term trends and the processes underlying the origin of biological complexity remain unresolved.

Long-term upward trends in complexity over evolutionary time have been hypothesized to be driven by external (e.g., environment, natural selection) and internal (e.g., development, natural variance) factors. External mechanisms can increase complexity through adaptive responses to direct or indirect natural selection, such as specialization for a new environment or behavior, or niche partitioning^4,8,9^. By contrast, internal drivers include increasing entropy^4,10^, or the so-called ‘zero force evolutionary law’ of ever-increasing variance, in which parts will naturally tend to differentiate from one another by random chance^11^. However, the concept of simple unidirectional long-term trends may belie a much more sophisticated suite of possible evolutionary patterns^12^. For example, non-equilibrium thermodynamics predicts that evolutionary systems can increase in complexity through time due to entropy, while simultaneously becoming organized due to phylogenetic, developmental, or environmental constraints^10^. Mechanisms invoking ‘attraction points’ for different clades can produce trend-like patterns by creating stepwise changes in evolutionary optima^13^. For instance, the ‘evolutionary ratchet’ hypothesis posits that stochastic jumps in complexity may accompany adaptive radiations into new niches and lay the groundwork for future increases^4,14^. Changing developmental constraints could produce a similar pattern. Although gene number itself is a poor correlate of complexity^3^, and thus likely does not constrain it, increasing genomic complexity in regulatory elements, such as those of the global patterning *Hox* clusters, can drive morphological innovation^3^. Therefore, increasing developmental modularity is hypothesized to drive complexity by expanding the potential for the generation of new morphologies and independent modules.

Testing hypotheses regarding complexity with biological data has proved challenging, in part because complexity itself is difficult to define and quantify. Complexity may be defined in terms of structures or processes, parts or interactions, within or between levels of biological organization^2,15–17^. Structural complexity, the focus of this work, is broadly defined as the degree of differentiation within a biological system^2,8,18^. In other words: how many distinctive parts are there and how different are they from one another? The simplest approach is to count numbers of discrete parts. For example, the number of cell types have increased through time in Metazoa in association with the origin of major clades, but subsequently plateaued within groups^3,19^. Opposite trends also exist, such as an overall reduction in the number of skull bones through time in vertebrates as a whole and particularly in the lineage leading to mammals^20^. However, counts are a poor measure of the functional differentiation of a system, a concept that is key to our intuitive understanding of complexity. Serially-homologous structures, such as limbs and vertebrae provide an opportunity to directly measure anatomical complexity because they contain repeating units whose differentiation can be compared^21^. For example, the degree of differentiation of limb-pair types (tagmosis) has increased through time in arthropods^18^. Complexity increases are correlated with species origination in multiple lineages of crustaceans, suggesting a long-term selection-driven trend in complexity^8^.

Taking these ideas further, McShea produced quantitative metrics for examining complexity in serially-homologous structures based on the vertebral column^21^. He defined the complexity of an organismic system as “the number of different parts it has, or the degree of differentiation among parts, and the irregularity of their arrangement”^7^. Thus, complexity refers only to the total differentiation of a system, not to the structure or function of its parts, and is reflected by their relative spread. However, complex biological systems intuitively incorporate the concept that parts must not only be differentiated from one another but must also be arranged so as to contribute to the function of the whole^1^. Therefore, we integrate complexity with a second concept—organization—defined by McShea as “the structuring of a system for some function, independent of the number of parts it has or its configurational heterogeneity”^7^. Here, we measure functional organization as the degree to which serially-homologous parts are structured in a non-random way, such as concentration about a mean shape, integration of neighboring parts in a series (‘order’ metrics of McShea^21^), or morphological clustering (Methods, Supplementary Fig. 1).

The mammalian vertebral column provides an ideal case study of a complex biological structure. Relative to most tetrapods, mammals display vertebral columns that are both complex and highly organized into distinctive morphological and functional regions (or modules), features that have been linked with enhanced locomotory capacity, respiratory efficiency, and endothermy in the mammalian lineage^22–25^. For example, vertebral specializations have been hypothesized to form part of a functional complex for increasing locomotor stamina in cynodonts, along with features such as the origin of the muscularized diaphragm in cynodonts^22,26^. However, the evolutionary mechanisms underlying the origin of this remarkable vertebral diversification are unclear. Ancestor-descendent comparisons in crown mammals revealed no consistent within-lineage increases in complexity, implying passive Brownian motion within the group^7^ and refuting expectations of a long-term active trend. If no increasing trend exists within crown mammals, this raises the question of when, how and by what mechanism vertebral complexity originated in the mammalian lineage. To address this question it is necessary to examine patterns in extinct members of the mammalian stem group, the non-mammalian synapsids^27^.

Here, we mathematically modeled vertebral evolution in a phylogenetic framework while integrating key data from the synapsid fossil record. Vertebral complexity and organization *sensu* McShea^7^ were analyzed in fifteen exceptionally-preserved non-mammalian synapsids and thirty-five extant mammals. Complete vertebral columns in the fossil record are rare, therefore we apply a novel Monte-Carlo simulation approach that uses a master phylogenetic tree (Supplementary Fig. 2) to address subsampling of taxa while statistically comparing evolutionary models in the context of the available sampling. Results indicate that increasing vertebral complexity and organization in synapsids is not gradual but is best described by a stepwise pattern of shifting adaptive optima. Correlations between vertebral metrics and basal metabolic rates in extant mammals, combined with the phylogenetic position of an inferred shift coincident with evidence of increased metabolic rate in non-mammalian cynodonts (e.g., muscular diaphragm, secondary palate), point to increasing aerobic capacity as the evolutionary driver of vertebral complexity in synapsid evolution.

## Result

### Evolutionary hypotheses

**Fig. 1:**
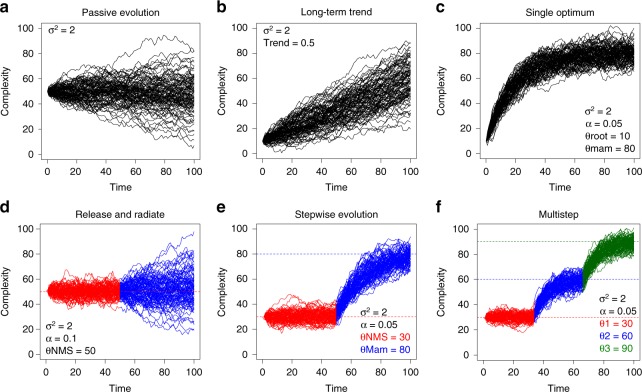
Hypotheses for the evolution of vertebral complexity in mammals. **a** Random walk (BM model); **b** long-term continuous upward trend (BMtrend model); **c** selection toward a high optimum value in mammals (rooted OU1 model); **d** constrained evolution in non-mammalian synapsids followed by unconstrained radiation in mammals (RR model); **e** stepwise evolution toward higher optimum in mammals (OU2 model). **f** Multistep evolution with multiple optima shifts (e.g., OU3A and OU3B)

**Fig. 2:**
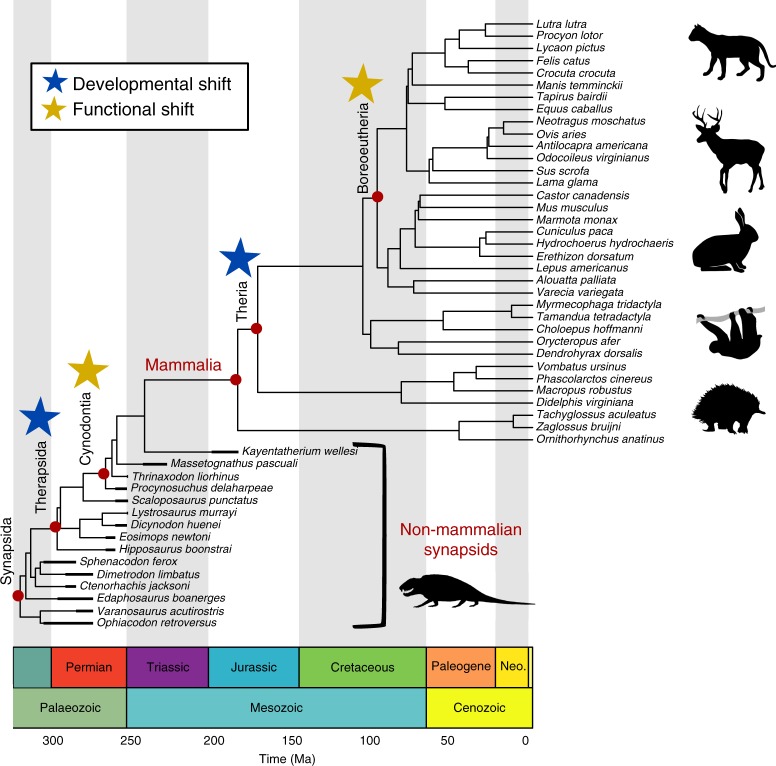
Time-calibrated phylogeny of sampled synapsid taxa. Thick lines: first and last occurrence dates of fossils. For more information see Supplementary Note 1. Non-mammalian synapsid divergence times reconstructed from ‘master’ phylogeny (Supplementary Fig. 9). Mammalian tree from Timetree.org^72^. Blue stars: Evolution of pectoral and ribless lumbar vertebral column regions occur at Therapsida and Theria^30^ respectively (OU3A model). Gold stars: Functional shifts inferred from increasing vertebral heterogeneity at Cynodontia and Boreoeutheria (OU3B model). Sloth image credit: Sarah Werning under, Creative Commons Attribution 3.0 Unported license, no changes were made

We explicitly test seven alternative hypotheses that could explain the origin of elevated vertebral complexity and organization in mammals (Fig. 1; Table 1): (1) Passive evolution, where increases in complexity are produced by random variation from a lower bound (Fig. 1a, Table 1: BM); (2) Active trend, where increases are due to an actively-driven long-term trend (e.g., increasing entropy or the ‘Zero force evolutionary law’^4^) (Fig. 1b, Table 1: BM trend); (3) Single optimum, an Ornstein-Uhlenbeck (OU) model^28^ with evolution toward an optimum in Mammalia from a different ancestral condition (separate root optimum) (Fig. 1c, Table 1: OU1); (4) Release and radiate, in which complexity is restricted in the synapsid stem lineage via an OU model, followed by Brownian motion reflecting a functional or developmental release in mammals^7,29^ (Fig. 1d, Table 1:RR); (5) Mammal shift, which invokes an OU model with a single regime shift at Mammalia associated with the origin of the crown group (Figs. 1e,  2, Table 1: OU2); (6) Developmental shift, which tests if complexity is constrained by the number of vertebral regions using an OU model with shifts in optima associated with the origin of the pectoral and lumbar regions at Therapsida and Theria^30^ (Figs. 1f,  2, Table 1: OU3A); and (7) Functional shift, which tests for stepwise increases in complexity associated with the evolution of region differentiation (heterogeneity) using an OU model with shifts at Cynodontia and Boreoeutheria^30^ (Figs. 1f,  2, Table 1: OU3B).

**Table 1:** Summary of evolutionary models

Name	Hypothesis	Description	Parameters
BM	Passive	Brownian motion	σ², θroot
BMtrend	Active trend	Brownian motion with a trend	σ², θroot, trend
OU1	Release and radiate	Rooted OU with single optima	σ², θroot, α, θopt
RR	Single optimum	OU -> BM at Mammalia	σ²OU, σ²BM,θ, α
OU2	Mammal shift	OU->OU at Mammalia	σ², θNMS, θMam, α
OU3A	Developmental shift	OU->OU->OU at Therapsida and Theria	σ², θNTS, θTherap,θTheria, α
OU3B	Functional shift	OU->OU->OU at Cynodontia and Boreotheria	σ², θNCS, θCyn,θBoreo, α

For shift locations see Fig. 2

### Vertebral complexity and organization

**Fig. 3:**
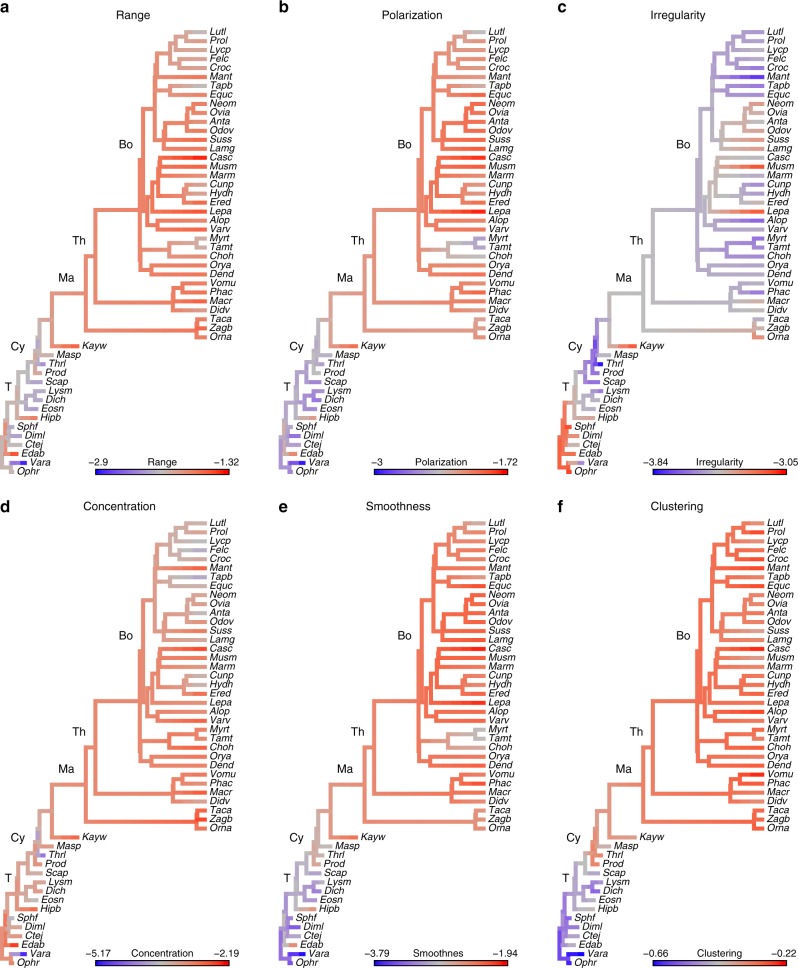
Variation in vertebral complexity and organization. Based on the whole vertebra dataset optimized onto the sampled synapsid phylogeny using maximum likelihood. Evolutionary patterns of complexity: Range (**a**), Polarization (**b**), Irregularity (**c**). Evolutionary patterns of organization: Concentration (**d**), Smoothness (**e**), Clustering (**f**). For abbreviations see Supplementary Table 7. T, Therapsida; Cy, Cynodontia; Ma, Mammalia; Th, Theria; Bo, Boreoeutheria

Vertebral complexity (range, polarization, and irregularity) and organization (concentration, smoothness, and clustering) were measured on the whole vertebra dataset, as well as two data subsets: centrum-only and arch-only. Values were optimized on the phylogeny using maximum likelihood to qualitatively examine patterns of variation in synapsid evolution (Fig. 3). Four metrics of complexity (degree of vertebral column differentiation) and organization (distribution of vertebrae along the column) showed an overall increase in synapsids, whereas one decreased then increased, and one showed no pattern, as described below.

Range and polarization (the spread of the data) are both generally lower in basal synapsids than in non-mammalian cynodonts and crown mammals, suggesting an overall increase through synapsid evolution (Fig. 3). The ‘pelycosaur’ *Edaphosaurus* also displayed relatively high values, relating to its exaggerated ‘sail’ consisting of elongated neural spines; high values were not recovered for the sail-backed *Dimetrodon*, presumably because the neural spines were excluded for the examined specimen due to damage. Xenarthrans displayed particularly low values. Irregularity (variation between adjacent vertebrae) did not support the hypothesis of evolutionary increase. Unlike the other two complexity metrics, ‘pelycosaurs’ exhibited the most irregular columns, followed by a decrease in non-mammalian therapsids and cynodonts, and a subsequent increase in some mammals (Fig. 3). Overall, patterns of complexity were similar in the centrum-only and arch-only subsets (Supplementary Figs. 3 and 4).

Both smoothness (similarity of adjacent vertebrae relative to non-adjacent vertebrae) and clustering (patchiness, or degree to which the distribution of the vertebrae deviates from a uniform distribution) increased in synapsids (Fig. 3, Supplementary Figs. 3 and 4), indicating that mammals have vertebral columns that cluster, but are integrated between adjacent vertebrae. Concentration (tendency of vertebral measurements to clump near the mean shape) varied little among synapsids (Fig. 3, Supplementary Figs. 3 and 4) indicating proportional changes in both range and polarization.

### Evolutionary models

**Fig. 4:**
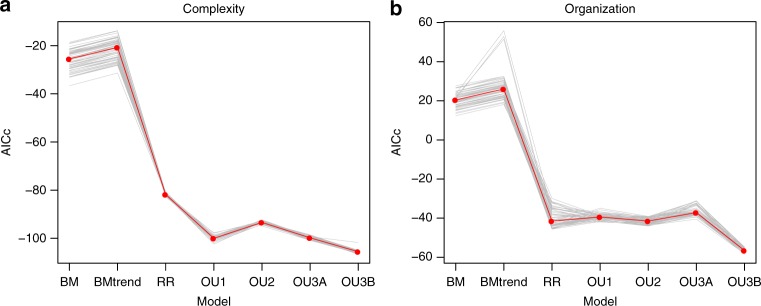
Comparison of model performance based on AICc. Models compared across 60 trees for vertebral complexity (**a**) and organization (**b**). Results based on whole vertebra dataset. For model definitions see Table 1. Source data are provided as a Source Data file

To test different evolutionary scenarios for producing mammalian vertebral complexity and organization, each of the seven evolutionary models were fit to the metrics across 60 phylogenetic trees reflecting variation in fossil branch length estimates. Based on the median AICc, the OU3B (Functional shift) model performed the best for both complexity and organization across all 60 trees (Fig. 4, Table 2), receiving strong support based on AICc weights (complexity: 0.89, organization: 1.0) and supporting stepwise evolution of these traits. The other models performed relatively poorly, particularly the BM and BMtrend models, indicating that long-term uniform trends do not provide a good fit for the data compared to other models tested here.

**Table 2:** Relative fit and rank order of evolutionary models for the whole vertebra dataset

	Rank	AICc	diff	wi	AICw
Complexity					
OU3B	**1**	**−105.6**	**0.0**	**1.00**	**0.89**
OU1	2	−100.0	5.6	0.06	0.06
OU3A	3	−99.8	5.8	0.06	0.05
OU2	4	−93.4	12.2	0.00	0.00
RR	5	−81.8	23.7	0.00	0.00
BM	6	−25.5	80.0	0.00	0.00
BMtrend	7	−20.7	84.8	0.00	0.00
Organization					
OU3B	**1**	**−56.5**	**0.0**	**1.00**	**1.00**
RR	2	−41.5	15.1	0.00	0.00
OU2	3	−41.5	15.1	0.00	0.00
OU1	4	−39.4	17.1	0.00	0.00
OU3A	5	−37.2	19.4	0.00	0.00
BM	6	20.5	77.0	0.00	0.00
BMtrend	7	26.0	82.6	0.00	0.00

Median values drawn from analysis across 60 phylogenetic trees

Bold: best model used for H1 in hypothesis test simulations

*AICc* Akaike information criterion, *diff* AICc difference, *wi* weighting, *AICw* Akaike weight

Optima values were estimated for OU models to provide information regarding evolutionary mode, and the median and confidence intervals were calculated by jackknifing (Supplementary Table 1). For all OU analyses, optima (θ) lay within the range of the observed data, rates (σ) were relatively high, and phylogenetic half-lives were short (Supplementary Tables 1–3). These results fit expectations of peak-type dynamics characterized by evolution toward an optimum value, as opposed to trend-like dynamics which are characterized by low rates and optima outside of the observed range.

In the best-fitting OU3B (Functional shift) model, optima differences were assessed based on the jackknifed confidence intervals. Significant increases between non-cynodont synapsids and cynodonts were observed for all measures except irregularity, for which there was a significant decrease (Supplementary Table 1). By contrast, significant increases were only observed for polarization and smoothness at the shift between non-boreoeutherian mammals and boreoeutherians. Results of between-group ANOVAs on the tip values confirm this result (Supplementary Table 4). There was a significant effect of grouping on range, polarization, smoothness, and clustering. Post-hoc Tukey HSDs revealed that there were significant differences for all the significant variables at the first shift, but only for polarization and smoothness at the second shift. Similarly, absolute values of increase were larger at the first shift than the second (Supplementary Table 1). Despite this result, additional analyses allowing a single shift at Cynodontia and a single shift at Boreoeutheria confirmed that the OU3B model fit the data better than either shift on its own (Supplementary Tables 5 and 6).

### Monte-Carlo simulations

**Fig. 5:**
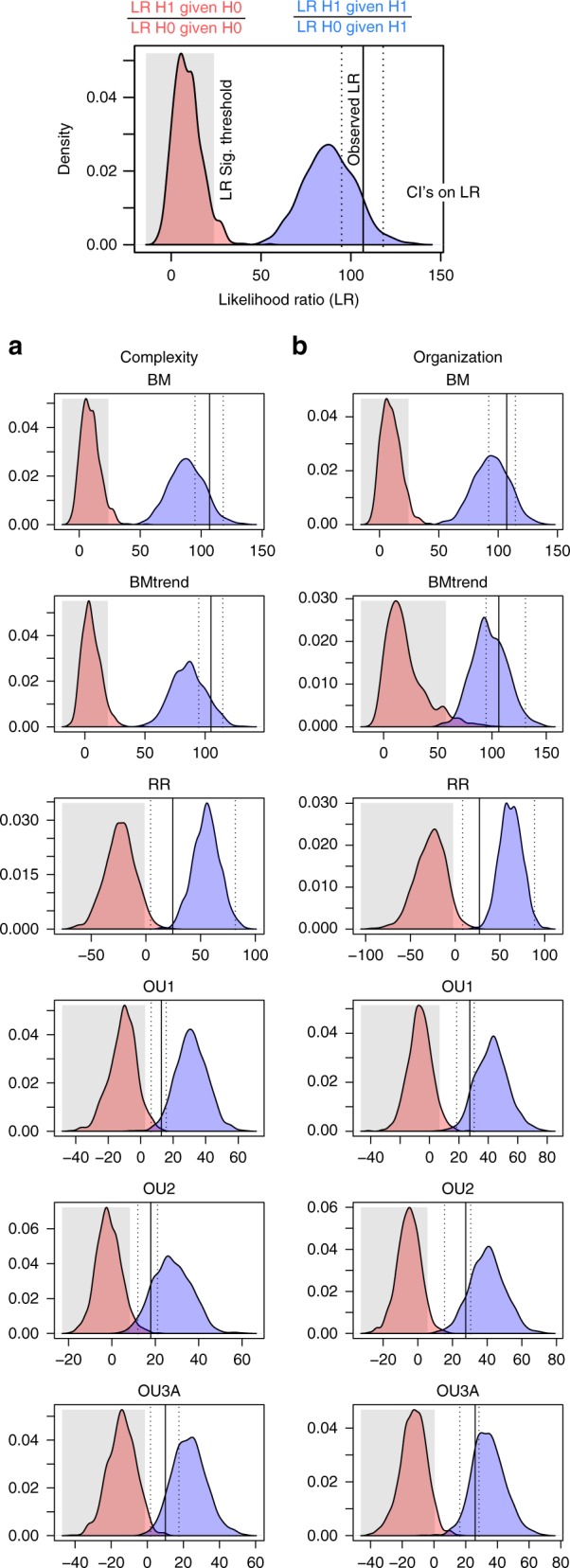
Hypothesis tests between the functional shift and other models. Based on 1000 simulations for complexity (**a**) and organization (**b**). Top figure is an example plot. Red curves represent simulated distribution of Likelihood ratio (LR) under the null model and blue curves represent the simulated distribution under the test model. Vertical line indicates median LR recovered from jackknifing the observed data, with dashed lines indicating their confidence intervals. The shaded box represents 97.5% of the simulated null distribution. H0 is rejected in favor of H1 if the observed LR falls outside the significance threshold based on simulation (shaded box). Source data are provided as a Source Data file

Simulations were performed to test for significant differences between the best-performing model and the other models given phylogenetic uncertainty and taxonomic sample (Fig. 5, Table 3). Each test compares the distribution of likelihood ratios (LR) generated under both the best model (Fig. 5: blue curve) and alternate model (Fig. 5: red curve) and compares the observed likelihood ratio (Fig. 5: ertical line) to the alternate model distribution. If the jackknifed confidence intervals (Fig. 5: dotted line) on the LR lie outside of the 95% confidence interval for the null distribution (Fig. 5: shaded area) there is strong support for rejecting the null model.

**Table 3:** Hypothesis tests of OU3B (H1) against various null models (H0) for the whole vertebra dataset

H0	H1	LnLik	LCI	UCI	Th	P-va
Complexity						
BM	OU3B	109.8	95.9	114.4	23.3	0.00
BMtrend		107.5	93.1	112.7	20.0	0.00
RR		23.7	5.9	81.6	−2.0	0.00
OU1		12.1	5.7	15.4	1.7	0.00
OU2		20.2	12.0	21.8	9.0	0.00
OU3A		5.8	2.2	16.9	−1.7	0.00
Organization						
BM	OU3B	107.0	92.6	114.9	24.1	0.00
BMtrend		105.6	89.2	129.1	58.4	0.00
RR		15.4	8.7	88.5	−1.5	0.00
OU1		25.2	16.0	30.4	5.7	0.00
OU2		23.0	14.1	30.1	5.8	0.00
OU3A		19.3	16.8	28.7	0.0	0.00

Log likelihood ratio tests (LR) based on Monte-Carlo simulations with taxonomic down-sampling and trees drawn at random from 60 phylogenies. LR are jack-knifed median values with 95% confidence intervals (LCI, UCI). Thresh: LR threshold required to reject the null hypothesis based on simulations

Simulations on the whole dataset strongly supported the OU3B (Functional shift) model, suggesting it is significantly preferred over all the other models, based on both the median observed LR (Fig. 5: black line) and its lower confidence interval (Fig. 5: dotted lines, Table 3). This approach considers both the topology and subsampling of the phylogeny. Therefore, these results suggest that the OU3B model is supported given the taxonomic sampling available.

## Discussion

**Fig. 6:**
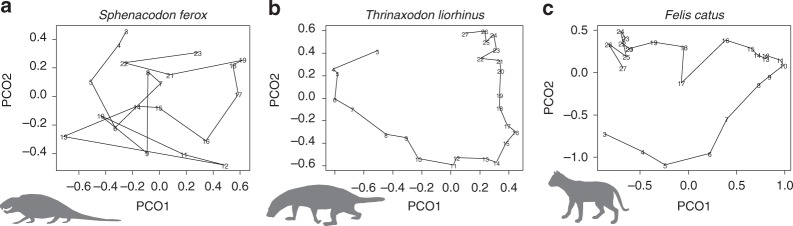
Comparative morphology of vertebral columns. **a** A ‘pelycosaur’, **b** a non-mammalian cynodont, and **c** a Boreoeutherian, illustrated using Principal Coordinates analysis. The vertebral columns of basal synapsids are relatively uniform (low range and polarization) but highly irregular. Through synapsid evolution, range and polarization increase, while columns become much smoother and more organized into clusters

Four out of six measures of complexity (range, polarization) and organization (smoothness, clustering) show an increasing trend in synapsids (Fig. 3), supporting previous qualitative and quantitative studies^30–33^. Range and polarization (reflecting differentiation of vertebrae), smoothness (reflecting integration between adjacent vertebrae), and clustering (reflecting modularity of vertebral regions) were generally higher in non-mammalian cynodonts and mammals than ‘pelycosaurs’ (Fig. 3). These results indicate that the degree of overall vertebral variation within each column increases while simultaneously becoming more organized as vertebrae are restructured into more distinct regions (Fig. 6). Thus, increases in variance in synapsids occurred in a highly-structured way and was not simply ‘scaled up’ in mammals. Further, differentiation of vertebrae in synapsid evolution did not occur at random with respect to position along the vertebral column. For example, smoothness is higher in mammals, suggesting that adjacent vertebrae are on average more alike relative to the mean, but clustering also increases, meaning that the distribution of vertebrae is uneven (Fig. 6). Taken together these results suggest both more integration between vertebrae within regions coupled with increased differences between regions in mammals^30^. One interesting caveat to this pattern is that it likely only applies to terrestrial mammals. Secondarily aquatic mammals such as whales and sirenians have highly uniform vertebrae, accompanied by loss of observable regions in some cases, associated with their derived ecology and mode of locomotion^34,35^. Although this study focuses on terrestrial mammals to provide a relevant comparison to non-mammalian synapsids, exploring the relationship between complexity and ecology within aquatic mammals will provide an interesting line of future inquiry.

Irregularity, which marks variation between adjacent vertebrae, decreases in synapsids (Fig. 3). The most basal synapsids, the ‘pelycosaurs’, have elevated irregularity confirming prior findings of high centrum irregularity in the group (Fig. 6). Based on a more limited sample, Felice and Angielczyk proposed that irregularity of the centrum in basal ‘pelycosaurs’ may reflect looser functional constraints between vertebrae due to the larger relative size and presumed functional role of the notochord^36^. Ancestrally, the notochord forms a significant portion of the vertebral column in early synapsids^31^, but the size and craniocaudal extent of the notochord is reduced through synapsid evolution, with only an embryological remnant in mammals^37^. In accordance with Felice and Angielczyk’s hypothesis, vertebral irregularity is reduced in non-mammalian therapsids and cynodonts that have a reduced notochordal contribution. However, analyzing the data subsets reveals that this pattern is manifest not only in the centrum that interacts directly with the notochord, but also in the arch measures, which might be hypothesized to be more independent of notochordal variation (Supplementary Figs. 3 and 4). This finding is supported by observations of alternating variation in neural spine height between adjacent vertebrae in some ‘pelycosaurs’, also implying irregularity of arch structures in basal synapsids^38^. Therefore, the notochord hypothesis may be insufficient to explain patterns of irregularity. Instead, there appears to be increasing between-vertebra constraints across the whole vertebra (centrum and arch) at the transition from ‘pelycosaurs’ to therapsids, possibly associated with concurrent changes in regionalization^30^. Concentration did not display any consistent trend, suggesting that the overall distribution of the vertebrae relative to the mean does not change systematically through evolutionary time. This indicates that increasing within-column disparity is driven by morphological divergence throughout the vertebral column, not by evolution of extreme morphologies of individual vertebrae or regions.

Passive diffusion and long-term driven trends cannot explain increasing complexity and organization in this dataset. Stephen J. Gould colorfully suggested that increasing complexity could be explained by a ‘drunkard’s walk’, whose path is hindered by a wall and therefore tends toward the opposite direction^6^. A key prediction of this hypothesis is that increases and decreases in complexity are equally likely at any given point in time^4,7,8^. We modeled this hypothesis using a Brownian motion process in which complexity and organization varied randomly in both magnitude and direction over any given interval, but it was rejected by both model-fit and simulation-based hypothesis tests (Tables 2 and 3, Figs. 4 and 5). The release and radiate model, which restricts Brownian motion to just the crown group after assuming an OU process in the stem group, resulted in a considerable improvement in model fit but was significantly less supported than models with multiple OU optima. These results hold in spite of the relatively limited sampling of complete vertebral columns available in the fossil record, as their significance is based on a Monte-Carlo simulation approach that accounts for sampling bias using subsampling during hypothesis testing (Methods). Therefore, a random walk is insufficient to explain the patterns of vertebral column complexity and organization in synapsid evolution.

Alternatively, a long-term active trend of increasing complexity may be driven by continuous selection for niche differentiation, increasing entropy, or the natural tendency for similar parts to diverge through evolution^4,11^. Active trends predict not only an increase in the mean and maximum total complexity across groups through geologic time (e.g., in metazoans^2,19^ and arthropods^18^) but also a tendency for values to increase within lineages and in parallel along branches of the phylogeny (e.g., in crustaceans^8^). If an active trend is driving mammal vertebral evolution, crown mammals should exhibit greater vertebral complexity than their non-mammalian synapsid ancestors. Furthermore, increases in complexity should be observed along the stem lineage and within sub-groups. Two findings show that the data do not support this hypothesis. First, the BMtrend model was poorly supported by both AICc and Monte-Carlo simulations, receiving even less support than a pure BM model (Tables 2 and 3). Second, OU models that fit the data well-recovered parameters for their optima within the range of the observed data (Supplementary Table 3). Trend-like dynamics are signified in OU models by low α and θ outside of the range of the data, representing slow evolution to a far-off optimum^13,39,40^.

Taken together, these data show that evolution of the mammalian vertebral column is not best explained by long-term actively-driven increases acting throughout the evolutionary history of synapsids. Our results instead support prior observations made within the mammalian crown group. In comparing extant mammals to a paired ‘underived sister taxon’ representing the ancestral condition, McShea found that increases and decreases in vertebral complexity occurred with equal frequency^7^. Although this approach is somewhat limited in its estimation of lineage-wise evolution (the degree to which the ‘underived’ taxa may represent the ancestral condition is debatable), it nevertheless highlights the absence of strong within-lineage trends in mammals despite elevated values relative to other major groups (i.e., when compared to fish or lizards as in ref. ^7^), implying discontinuous change in different clades and periods of geologic time.

Instead of long-term, uniform trends, the data presented here most strongly support stepwise evolution of synapsid vertebral complexity and organization, indicating that evolutionary patterns vary across groups and through time. Similar stepwise evolutionary patterns have also been described for body size in archosaurs, dinosaurs, and pterosaurs—another trait that has traditionally been thought of as displaying a long-term active trend (‘Cope’s rule’)^13,39,40^.

Stepwise trait evolution could be the result of selection, the release of constraints, or a combination of both processes. In a constraint-driven process, stepwise patterns can be generated in a system in which there is an underlying long-term trend (such as increasing entropy^10^) by the sequential addition or removal of functional or developmental constraints that limit the generation of complexity. In this case, the transition between steps is marked by the evolution of novel phenotypes or developmental mechanisms that provide new opportunities to generate more complex morphologies. In a selection-driven process, stepwise patterns are instead generated by adaptation to novel selective regimes or release of functional constraints, relating to transitions in behavior, ecology, or functional complexity. An example of this type of mechanism would be the ‘ratchet hypothesis’ proposed by Stebbins, in which strong selection for complex morphologies occurs during adaptive radiations into new ecological niches, producing an evolutionary ‘ratchet’ of increasing complexity in successive clades^14^. These two frameworks provide the end points of a continuum of evolutionary scenarios in which the generation of or selection for phenotypes may cause heterogeneity in evolutionary patterns or may combine in various ways through the evolutionary history of a clade.

We tested the role of developmental constraint using the Developmental shift (OU3A) model, where the evolution of vertebral regions drives increasing complexity and organization. Modularity has been argued to be “one of the most critical features underlying the evolution of large and complex animals”, and a driver of phenotypic and taxonomic diversity^3^. Conversely, strong integration of serially-homologous structures through their development and evolution may act to restrict the generation of novel morphologies that can produce vertebral complexity. In the vertebral column, the expression of regionalizing *Hox* genes mark boundaries between phenotypically distinctive vertebrae and has been implicated in driving modularity^41,42^. Likewise, in arthropods the evolution of *Hox* regulation along the anteroposterior axis and within-limb hierarchies has been proposed to have driven diversification of segment types associated with evolutionary increases in limb complexity^3,8,18^. Mammal vertebral columns are strongly modular^42,43^, and our recent work suggests that regionalization increased during synapsid evolution, implying increasing modularity through time^30^. The Developmental shift model provided a relatively good fit to the data, but it was significantly less supported than the preferred model for both complexity and organization. Therefore, the evolution of new regions does not seem to be the trigger for increasing vertebral complexity and organization.

Instead, our results support the Functional shift (OU3B) model, where optima shifts are associated with increasing heterogeneity of the vertebral column. Vertebral heterogeneity in mammals, or the morphological differentiation of the vertebral column among regions, has been linked to functional specialization of vertebral regions^44–47^, as well as ecomorphological diversification and increased rates of evolution^43,48^. Therefore, this result supports the independence of the generation of regions from their morphological and functional differentiation, mimicking patterns found in the diversification of limb appendages in insects^49^. In other words, developmental modularity may be prerequisite for vertebral complexity, but an additional selective trigger may also be required to realize vertebral diversity. Optima values suggest that the evolution of the synapsid vertebral column is characterized by a major shift in complexity and organization at Cynodontia in the late Permian, followed by a smaller increase in the Cretaceous within the northern placental mammals, the Boreoeutheria. Based on the phylogenetic location of these shifts and their correlation with various physiological and functional traits, as outlined below, we hypothesize that adaptation for increasing aerobic capacity in synapsids likely drove this stepwise pattern.

Mammals are endothermic, meaning they can metabolically produce body heat, a trait associated with sustained activity and locomotor stamina via enhanced aerobic capacity^50,51^. Whereas ectotherms such as lizards perform well over short bursts, mammals can maintain much higher activity levels over long periods. In terms of locomotion, mammals can maintain sustained running speeds of around eight times that of a comparable lizard^51,52^ and use their higher stamina to forage widely^53^. Understanding the evolutionary history of mammal endothermy is complicated because many associated traits are intercorrelated and difficult to trace in the fossil record^50,51^. However, aerobic capacity for enhanced activity levels and locomotor stamina is one hypothesized selective factor for increasing basal metabolic rate (BMR)^52^. Although the precise evolutionary timing of mammalian endothermy remains controversial, the shift to a more active lifestyle is linked with numerous musculoskeletal specializations^50^, many of which can be identified in non-mammalian cynodonts (see below).

Carrier^22^ directly implicated the vertebral column in adaptation for aerobic capacity as part of a functional complex whose role was to circumvent an ancestral locomotor constraint in tetrapods – conflicting use of the axial musculature in both locomotion and respiration (Carrier’s constraint)—thus allowing mammals to move and breathe at the same time. These traits include a muscular diaphragm to free the body wall of ventilatory requirements, axial modifications that stabilize against lateral motion or provide increased independence of the vertebral musculoskeletal system from that of the ribs, and asymmetric mammalian gaits with sagittal bending that can facilitate aspiration^22^. Released from this respiratory constraint, the mammalian vertebral column may have been free to diversify, enabling specialization of particular regions for new functions (e.g., sagittal bending occurs only in the posterior dorsal joints^25^), driving increases in vertebral complexity and organization.

The largest optima shift in complexity and organization (indicating attraction toward a higher value) was recovered at Cynodontia and is consistent with multiple lines of fossil evidence that, taken together, point towards increased aerobic capacity in this group^50,51^. For example, the origin of the muscular diaphragm is a key marker of locomotory-respiratory decoupling and ventilatory efficiency. While pectoral region differentiation associated with forelimb postural shifts in early therapsids may have provided the impetus for thoracic wall muscularization^30^, fixation of the cervical count at seven implies the muscularized diaphragm did not arise until within cynodonts (e.g., *Thrinaxodon*^26,54^). The evolution of respiratory turbinates, a mammalian feature associated with increased lung ventilation and body temperature, is inferred from bony ridges in the nasal cavity in both therocephalians and cynodonts, likely convergently^50,51,55,56^; and the Early Triassic cynodont *Thrinaxodon* possessed a wide mammal-like nasal passage suitable for housing fully-developed turbinates^50^. Non-mammalian cynodonts are also among several synapsid groups to evolve a secondary palate^51,55^, a bony plate that divides the oral and nasal cavities linked with increased ventilation rate, and oxygen isotope data provide strong evidence of elevated body temperature among advanced cynodonts^57^. Thus, multiple lines of evidence point to elevated activity levels and aerobic capacity in non-mammalian cynodonts.

The second, subtler, shift recovered in Boreoeutheria likely reflects a similar relationship with aerobic capacity via the repeated evolution of highly-aerobic cursorial specialists within the northern placental mammals. Among mammals, Boreoeutheria contains most extant diversity (around 5000 species), including ecologically-specialized clades^58^, as well as all living cursorial placentals (e.g., horses, antelope, rabbits). Both running speed and maximal metabolic rate correlate with BMR^59^, and body temperature correlates with metatarsal to femur ratio, a proxy for cursoriality^60^, suggesting that high-stamina cursorial locomotion is an important driver of aerobic capacity. Whereas southern placental mammals (i.e., members of Atlantogenata) are characterized by lower BMR^61^, lower body temperatures^62^, and high developmental lability^63,64^, boreoeutherians exhibit high developmental stability and pulses of ‘supraendothermy’ (body temperature above 38 degrees) associated with the evolution of cursoriality in numerous families^60^.

To further test the aerobic capacity hypothesis, we gathered data on crown mammal BMR and body temperature—physiological proxies of aerobic capacity—and examined their correlation with our vertebral complexity and organization measures. Multivariate regressions revealed highly-significant correlations between both physiological parameters, complexity (BMR: Pillai’s *p* = <0.001; temp: Pillai’s p = 0.001) and organization (BMR: Pillai’s *p* = <0.001; temp: Pillai’s *p* = <0.001). When phylogenetic correlations between taxa were incorporated using Phylogenetic generalized least squares (PGLS), correlations with complexity remained significant (Supplementary Fig. 5, Supplementary Table 11, BMR: *p* = 0.019, rsq = 0.13; temp: *p* = 0.005, rsq = 0.22), whereas those with organization were marginally insignificant (BMR: *p* = 0.13, rsq = 0.08; temp: *p* = 0.065, rsq = 0.15). Therefore, data from extant species support a link between aerobic capacity and vertebral complexity in synapsids.

Evolutionary increases in morphological complexity have been widely discussed in the literature, yet empirical data testing these patterns are scarce. Using a dataset of exceptionally-preserved fossils and extant specimens, we tested competing hypotheses for the evolution of vertebral complexity and organization in synapsids. We reject the hypotheses of random passive increases or long-term evolutionary trends for increasing complexity. Instead, our data support stepwise evolution of complexity and organization via multiple evolutionary optima, suggesting clade-specific adaptations influenced complexity through geologic time. Stepwise evolution may represent increasing modularity coupled with an active trend, selection for stepwise adaptive optima relating to the evolution of novel mammalian vertebral functions, or a combination of both processes. Our results suggest that shifts in complexity did not coincide with the evolution of morphological regions, implying that developmental constraints are not limiting the evolution of complexity in synapsids. Instead, our data support a large shift in complexity and organization occurring at Cynodontia, followed by a smaller shift in Northern placental mammals (Boreoeutheria). Timing of the inferred evolutionary shifts, combined with fossil evidence and the correlation of complexity with BMR and body temperature in mammals, support aerobic capacity as a selective driver of stepwise patterns in synapsids. Thus, selection for higher activity levels combined with the release of respiratory constraints in cynodonts may have provided the trigger required to achieve vertebral complexity, and the subsequent biomechanical and ecological diversification of the presacral column in mammals^25,43,44^.

## Methods

### Sample

The sample included a broad taxonomic range of extant mammals (*n* = 35) and exceptionally-preserved fossil non-mammalian synapsids (*n* = 15) selected to encompass major stages in synapsid evolution, including ‘pelycosaurs’, basal therapsids, and cynodonts (Fig. 2, Supplementary Table 7). All specimens were adult based on size and epiphyseal fusion and extant taxa were chosen to span a broad range of terrestrial ecologies across the mammalian phylogeny. Highly derived aquatic or flying taxa were not included as such specialized morphologies are not central to the synapsid-mammal transition. Non-mammalian mammaliaforms were not included due to the lack of appropriately-preserved three-dimensional material but the effect of excluding these taxa was considered in the subsampling simulations described below. The vertebral columns of all fossil specimens were complete or nearly complete (over 85% mean completeness, see ref. ^30^), with minimal distortion.

### Measurements

Fifteen linear measurements from all post-axial, pre-sacral vertebrae were taken from Jones et al.^30,65^ (Supplementary Table 8). The measurement protocol captured variation in the centrum, neural arch, zygapophyses, and muscular processes. Transverse processes were broadly defined to include cervical transverse processes, diapophyses, and lumbar transverse processes. Mammal vertebrae are composed of two major ossification centers, the centrum (formed from the ancestral tetrapod pleurocentrum) and the neural arch, which have been hypothesized to form evolutionary modules^66^. To examine variation across these modules, the evolutionary patterns of complexity and organization were compared centrum-only and arch-only data subsets (Supplementary Table 8). The pleurocentrum and neural arch are the dominant centers of ossification in non-mammalian synapsids as well. Intercentra are retained in “pelycosaurs” and some therapsids (e.g., refs. ^67,68^), but they are very minor components of the vertebral column with simple morphologies, and we excluded them from our analyses^67,68^. Missing data were recorded as NA and were excluded in complexity and organization calculations. Following the previous studies^7,36^, data were logged prior to analysis to enable comparison of variance within each column across specimens of different sizes. All analyses were conducted in R^69^.

### Metrics of complexity and organization

For each specimen, three metrics of vertebral complexity (range, polarization, and irregularity) and three metrics of organization (concentration, smoothness, and clustering) were calculated^7,21,36^. Complexity metrics measure the total differentiation of the vertebral column, whereas the organization metrics quantify relative distribution of the vertebrae. Metrics were calculated following McShea^21^ with the addition of the clustering metric, but applied in a multivariate fashion over all the logged linear data.

Range is the maximum spread of the data and was calculated as the maximum multivariate Euclidean distance (ED). The ED between vertebrae i and h is calculated over *p* linear measures as in Eq. (1).

1 $$ED_{{\mathrm{i}},{\mathrm{h}}} = \sqrt {\mathop {\sum }\limits_{{\mathrm{j}} = 1}^{\mathrm{p}} (x_{{\mathrm{i}},{\mathrm{j}}} - x_{{\mathrm{h}},{\mathrm{j}}})^2}$$

where *x*_*i,j*_ is the *j*th measurement taken from the *i*th vertebra and *x*_*h,j*_ is the *j*th measurement from the *h*th vertebra.

Polarization is the average spread of the data from the mean and was calculated as twice the mean Euclidean distance from the mean shape, $\bar x$, calculated using Eq. (2).

2 $${\mathrm{Polarization}} = 2\mathop {\sum }\limits_{{\mathrm{i}} = 1}^N ED_{{\mathrm{i}},\bar x}/N$$

Irregularity is the average spread of the vertebrae from their neighbors and was calculated as the average Euclidean distance between adjacent vertebrae.

3 $${\mathrm{Irregularity}} = \mathop {\sum }\limits_{{\mathrm{i}} = 1}^{N - 1} ED_{{\mathrm{i}},{\mathrm{i}} + 1}/(N - 1)$$

All three complexity metrics were scaled by the total number of linear measures, P, for each specimen. This step was unnecessary for the organization metrics because complexity measures were already corrected, and clustering is unitless.

Concentration measures the degree to which the vertebrae clump toward the middle of the distribution versus occupying extreme morphologies.

4 $${\mathrm{Concentration}} = {\mathrm{range}} - {\mathrm{polarization}}$$

Smoothness of the gradient along the column reflects the degree of integration between adjacent vertebrae.

5 $${\mathrm{Smoothness}} = {\mathrm{polarization}} - {\mathrm{irregularity}}$$

Clustering is the relative deviation of the data from a uniform distribution and was measured using the Hopkins Statistic, H. This compares the nearest neighbor distances of the data (*x*_i_), to nearest neighbor distances from a randomly-simulated uniform distribution (*y*_1_).

6 $$H = \frac{{\mathop {\sum}\nolimits_{{\mathrm{i}} = 1}^n {x_{\mathrm{i}}} }}{{\mathop {\sum}\nolimits_{{\mathrm{i}} = 1}^n {y_{\mathrm{i}}} + \mathop {\sum}\nolimits_{{\mathrm{i}} = 1}^n {x_{\mathrm{i}}} }}$$

where H measures the uniformity of the data. Thus clustering is defined as 1-H. It was calculated using ‘get_clust_tendency’ in the package ‘factoextra’^70^. The function was modified to permit missing data but exclude missing values from distance calculations (for more information see stats::dist).

### Sensitivity analyses

To understand the impact of missing data and presence/absence data on our results, we conducted two sensitivity analyses. First, the influence of missing data on the complexity and organization metrics was examined by simulating missing data. For three complete specimens (*Mus musculus, Tachyglossus aculeteus, Thrinaxodon liorhinus*), data were randomly removed and metrics recalculated across 100 iterations. Data were removed element-wise (missing measurement), row-wise (missing vertebrae) and column-wise (missing variable), at 10, 20, and 30% levels (Supplementary Note 2, Supplementary Fig. 6).

In mammal vertebral columns, some structures may be variably present or absent on different vertebrae along the column. For example, transverse processes are commonly absent on posterior thoracic (post-diaphragmatic) vertebrae. These absent structures were coded as missing (NA) in the main analysis to facilitate calculation of the above complexity and organization metrics. To examine how absent structures impacted the metrics, a second sensitivity analysis was conducted to assess the potential effect of excluding these serial absent structures (Supplementary Note 3). Absent structures were included in the sensitivity analysis using two approaches: data were scaled to mean centrum length and absent structures were coded as a small number (0.01) following^71^ (‘AbsSmall’ dataset) or they were coded as zero and an arbitrary unit of one was added to the whole dataset (‘AbsOne’ dataset) (Supplementary Note 3, Supplementary Figs. 7 and 8, Supplementary Tables 1 and 2).

### Phylogenetic trees

To ensure proper node-date estimation given the limited fossil sampling, a time-scaled phylogeny of the sampled taxa (sample tree: Fig. 2) was generated by subsampling a much larger synapsid phylogeny (‘master’ tree: Supplementary Fig. 2). The master tree was constructed as a composite phylogeny from three sources. First, positions and branch lengths of the 35 sampled extant species were taken from ‘timetree.org’^72^, which constructs relationships based on comparisons of all published molecular phylogenies of the taxa. Second, 56 fossil mammaliaforms and crown mammals were added at positions suggested by Slater^29^ based on combined molecular and morphological data. Branch lengths of the mammaliaform topology were scaled to time based on occurrence data from the paleobiology database (www.paleobiodb.org), using the packages ‘velociraptr’ and ‘paleotree’ and the function ‘timePaleoPhy’ using a minimum branch length of 1ma^73^. Finally, an extensive phylogeny of 353 non-mammalian synapsids was constructed from several phylogenetic analyses of individual synapsid clades (e.g., refs. ^74–83^, but see Supplementary Note 1 for all sources). Time bins for terminal taxa at the resolution of geologic stage were gathered from Brocklehurst^84^ and other literature sources, and the paleobiology database (Supplementary Note 1). A population of 60 phylogenetic trees was then generated from ‘bin_timePaleophy’ using the minimum branch length method, in which ‘true’ first and last occurrence dates are randomly sampled from within their known time bin^73^. The time-scaled mammaliaform phylogeny was bound to the non-mammalian synapsid phylogeny at the common taxon *Sinocondon rigneyi* to produce 60 master trees with 444 tips. These master phylogenies were finally subsampled to produce 60 sample trees (50 tips) with varying stem node ages (e.g., Fig. 2). Variation of the metrics was visualized by mapping them onto the phylogeny using maximum likelihood with the ‘contMap’ function of ‘phytools’^85^.

### Evolutionary modeling

Six different multivariate evolutionary models were fit to the complexity and organization data separately using the R package ‘mvMORPH’^86^ (Table 1). Shifts were specified on the phylogeny by creating a SIMMAP tree using ‘paintSubTree’ function in the package phytools^85^. Alpha and sigma matrices were unconstrained, allowing for trait correlation. Model input parameters were as follows:

1. BM—Brownian motion with no trend. Function: ‘mvBM’, model: ‘BM1’.
2. BMtrend—Brownian motion with increasing mean. Function: ‘mvBM’, model: ‘BM1’, param: ‘trend = T’.
3. OU1—Selection toward a single optimum from lower root value. Function: ‘mvOU’, model:’OU1’, param: ‘root = T’.
4. Release and radiate (RR)—Constrained evolution (OU) followed by release (BM). Function: ‘mvSHIFT’, model: ‘RR’.
5. OU2—Shift of optima at base of mammals. Function: ‘mvOU’, model: ‘OUM’, param: ‘root = F’.
6. OU3A— Two shifts at Therapsida and Theria. Function: ‘mvOU’, model: ‘OUM’, param: ‘root = F’.
7. OU3B —Two shifts at Cynodontia and Boreoeutheria. Function: ‘mvOU’, model: ‘OUM’, param: ‘root = F’. Given the support for this model, an additional analysis was also conducted that included two additional models with shifts occurring at Cynodontia and Boreoeutheria separately.

Models were fit across the 60 sample trees and the median values taken. Fit of the models was first assessed using the Akaike Information Criterion, corrected for small sample sizes (AICc), from which relative Akaike weights of the various models could be compared^86^. Significance of the differences between optima values was assessed using jackknifed confidence intervals (see below) and Analysis of Variance with post-hoc Tukey HSD on the tip data (r functions: *aov, TukeyHSD*).

### Monte-Carlo simulations

Monte-Carlo simulations of likelihood ratios were used to conduct pairwise hypothesis tests between specific models^87^. Due to the computational demands of the analysis, the best-fitting model based on AICc was included as the test model (H1) and compared iteratively to the other less-supported models (H0). The approach taken was based on that applied by Boettinger et al., but applied multivariately^87^. It quantifies the power and significance of the fit of a test model (H1, in the main dataset:OU3B) over a null model (H0) by simulating data under both scenarios and comparing them. Data were simulated 1000 times under four scenarios: the H0 model was run with data generated under the H0 model; the H1 model was run with data generated under the H0 model; the H0 model was run with data generated under the H1 model; and the H1 model was run with data generated under the H1 model. From these simulated likelihood values, likelihood ratios were generated between the H0 and H1 models under both a H0 and H1 scenario^87^. The distribution of these ratios was then compared to assess the relative power of the test, given the phylogeny. P-values were calculated by comparing the observed likelihood ratio between H0 and H1 models with the distribution of ratios generated under the H0 scenario.

Measuring vertebral complexity and organization requires very high skeletal completeness. Therefore, sampling potential among fossil taxa is limited. The Monte-Carlo simulations were further modified to simultaneously examine the impact of down-sampling on model selection, bias due to phylogenetic structure, and the potential impact of uncertainty associated with the dating of nodes. Data for each of the 1000 simulations were simulated on the more comprehensive master tree (444 tips, see above) but were repeatedly down-sampled to the sample tree (50 tips) prior to model fitting. To incorporate phylogenetic uncertainty, the tree used for the simulations was drawn at random from the available population of 60 phylogenetic trees for each simulation. To examine the influence of sampling on the observed likelihood ratio and optima values, a median likelihood value and confidence intervals were calculated by jackknifing the taxa while sampling across the 60 phylogenies.

The function ‘pmc’ for Monte-Carlo simulation hypothesis testing of evolutionary models^87^ was modified to include multivariate data (using ‘mvMORPH’ models^86^), phylogenetic uncertainty, and down-sampling as described above in a new function ‘mv_pmc_multiPhylo’ available at github: https://github.com/katrinajones/Functions.

### Multivariate regressions with BMR and body temperature

To test for a link between increasing complexity and organization and aerobic capacity in crown mammals, multivariate regression with physiological indicators of aerobic capacity was used. Basal metabolic rate (BMR) and resting body temperature were gathered from the literature for species or genera overlapping with our study^61^. BMR is correlated with maximal metabolic rate, heart-rate, ecology and locomotor performance in mammals^50,59,88^, whereas body temperature is correlated with aerobic scope, ATP generation by mitochondria and muscle-power output^89^, making these measures useful proxies for aerobic capacity and stamina. BMR is strongly related to body size, so size was removed prior to analysis by taking the residuals from a log-log regression against body mass from the literature, including a quadratic term as recommended in ref. ^61^. BMR and temperature data were available for 23 and 18 of the mammal species examined in this study, respectively. An additional four and two taxa respectively were available for different species within the same genus and so, to maximize sampling, the genus mean was used. Multivariate regressions were performed on the six complexity or organization metrics against BMR and body temperature and significance were tested using the Pillai’s trace statistic. To account for the impact of phylogenetic signal on the relationship between the variables, phylogenetic generalized least squares (PGLS) was conducted using the ‘ProcD.pgls’ function in the package ‘geomorph’^90^. This approach uses randomization of model residuals, permuted across the tips of the phylogeny, to test the significance of multivariate relationships in a non-parametric framework^91,92^. Relationships were visualized using the regression scores, from the ‘plot.ProcD.lm’ function, that represent the component of variation most highly correlated with the independent variable^93^.

### Reporting summary

Further information on research design is available in the Nature Research Reporting Summary linked to this article.

## Supplementary information

Supplementary Information

Reporting Summary

Source Data

## Data Availability

Data required to replicate this study are available in Dryad and include raw measurements, complexity measures, fossil time ranges, and phylogenies. Raw measurements: 10.5061/dryad.jm820mg; other data: 10.5061/dryad.5mkkwh71h. The source data underlying Figs. 4 and 5 and Supplementary Figs. 6 and 9 are provided as a Source Data file.

## Code availability

R functions used for Monte-Carlo simulations (‘mv_pmc_multiPhylo’) and to calculate the Hopkins Statistic with missing data (‘get_clust_tendency’) are available at github https://github.com/katrinajones/Functions.
