# Supplementary material for: Stepwise shifts underlie evolutionary trends in morphological complexity of the mammalian vertebral column
Source: Nat Commun. 2019 Nov 7;10:5071. doi: 10.1038/s41467-019-13026-3 (PMC6838112; doi:10.1038/s41467-019-13026-3)
Supplement: Supplementary file 1 — Supplementary Information [file 41467_2019_13026_MOESM1_ESM.pdf]

SUPPLEMENTARY INFORMATION

Stepwise shifts underlie evolutionary trends in morphological complexity of the mammalian vertebral column

Jones et al.

Supplementary Notes

Supplementary Note 1: Construction of the non-mammalian synapsid phylogeny p2

Supplementary Note 2: Sensitivity to missing data p2

Supplementary Note 3: Sensitivity analyses to absent serial structures p3

Supplementary Figures

Supplementary Figure 1-9 p4-12

Supplementary Tables

Supplementary Tables 1-11 p13-24

Supplementary References p25-30

Supplementary Notes

Supplementary Note 1: Construction of non-mammalian synapsid phylogeny

There is no single comprehensive phylogeny available for non-mammalian synapsids. Instead, nearly all phylogenetic research had focused on relationships with individual sub-clades. Therefore, we constructed a composite phylogeny by grafting together trees gathered from a variety of literature sources into a supertree (Supplementary Figure 2). The topology for pelycosaur-grade synapsids is primarily based on the reduced strict consensus of Brocklehurst et al.[1], with additional details on edaphosaurids added from Modesto[2], and details on sphencodontids (including the placement of *Tetraceratops* and *Raranimus*) based on strict-consensus tree of Brink et al.[3]. The backbone topology for higher level therapsid relationships is based on Sidor and Hopson[4]. Biarmosuchian relationships are based on Day et al.[5]. The topology for anteosaurid dinocephalians is from Kammerer[6] with *Pampaphoneus* added from Cisneros et al.[7]. The position of *Estemmenosuchus*, *Styracocephalus*, and the titanosuchids are based on Rubidge and van den Heever[8]. The phylogenetic relationships of tapinocephalid dinocephalians have not been examined in detail, so terminal taxa we added in a polytomy following the taxonomy of Atayman et al.[9] and Güven et al.[10]. The topology for Anomodontia is from Angielczyk and Kammerer[11], with the position of *Abajudon* following Olroyd et al.[12]. The topology of Gorgonopsia is from Kammerer and Masyutin[13]. The topology for Therocephalia is a hybrid incorporating the new taxa described by Kammerer and Masyutin[14] in the more highly resolved tree of Huttenlocker and Smith[15]. The phylogeny of non-mammalian cynodonts incorporates information from several analyses of varying scope. Basal cynodont relationships are based on Van den Brandt and Abdala[16]. The topology for Cynognathia is a hybrid of the analyses of Ray[17] and Pavanatto et al.[18]. The main topology for Probainognathia is a hybrid of Ruta et al.[19], Martínez et al.[20], and Martinelli et al.[21]. Details of tritylodontid and tritheledontidae are based on Velazco et al.[22] and Oliveira et al.[23], respectively.

To time-scale the tree we compiled stage-level stratigraphic range information from a variety of sources. A large number of ranges were taken from Appendix M of Brocklehurst[24], with additional range information gathered from other literature sources[3, 5, 6, 11, 12, 13, 14, 15, 16, 17, 18, 20, 21, 24, 25, 26, 27, 28, 29, 30, 31, 32, 33, 34, 35, 36, 37, 38, 39, 40, 41, 42, 43, 44, 45, 46, 47, 48, 49, 50, 51, 52, 53, 54]. A small number of ranges were taken from the paleobiology database (www.paleobiodb.org). Taxa were assigned to the stage(s) of the geological time scale in which they occur, and the numerical ages for each taxon's calibration are the ages of the beginning and the end of the stage(s) in question on the International Chronostratigraphic Chart (v2019/5[55]). Time ranges used to scale the phylogeny, including their literature sources, are available through Dryad (https://doi.org/10.5061/dryad.5mkkwh71h).

Supplementary Note 2: Sensitivity to missing data

To examine the influence of missing data in the fossils on the estimation of complexity and organization metrics, a sensitivity analysis was conducted. Results can be found in Supplementary Figure 6. Missing data up to 30% had a very minor effect on the estimation of five out of six of the metrics. The largest effect was on clustering, which appeared to be sensitive to data removal in mammals. However, the effect was small relative to the range of observed values among species (<0.05 vs ~0.4), suggesting that biological differences could still be detected.

Supplementary Note 3: Sensitivity analyses to absent serial structures

Anatomical structures which are present on some, but not all vertebrae, present an analytical challenge. Though presence and absence of structures is an important component of variation and thus complexity, incorporating this into traditional metrics of variance can be difficult. For the whole vertebra dataset, absent serial structures were excluded from the complexity and organization analyses by coding them as 'NA' (missing data). To estimate the potential effects of excluding absent structures on our results, we ran two sensitivity analyses: in the 'AbsSmall' dataset all data were scaled to mean centrum length then absent structures were coded as a small number (0.01); in the 'AbsOne' dataset they were coded as zero and an arbitrary unit of one was added to the whole dataset, prior to logging the data.

When absent structures were included similar overall patterns were observed to the main dataset for range, polarization, concentration, smoothness and clustering (Figure 3, Supplementary Figures 7-8). However, patterns of irregularity varied between the datasets. Whereas irregularity patterns observed in the AbsOne dataset resembled that of the main dataset, an overall increase in irregularity occurred when the AbsSmall dataset was used. Because serial absence of structures is primarily observed in mammals (e.g., transverse processes in posterior thoracic region), these results suggest that excluding them may underestimate irregularity in mammals. Given that serial absent structures contribute to vertebral irregularity in mammals, estimates that ignore this variation may be considered conservative.

Irrespective of how serial absent structures are treated, a stepwise evolutionary pattern is supported. The best-supported model, based on AIC, is still OU3B in all cases except complexity for AbsOne, in which case it is the second-best model after OU1 (Supplementary Figure 9, Supplementary Table 9). Further, the OU3B model is supported for both AbsOne and AbsSmall, for complexity and organization, based on comparisons of Monte-Carlo simulations with the median observed likelihood ratio (Supplementary Table 10).

Supplementary Figures

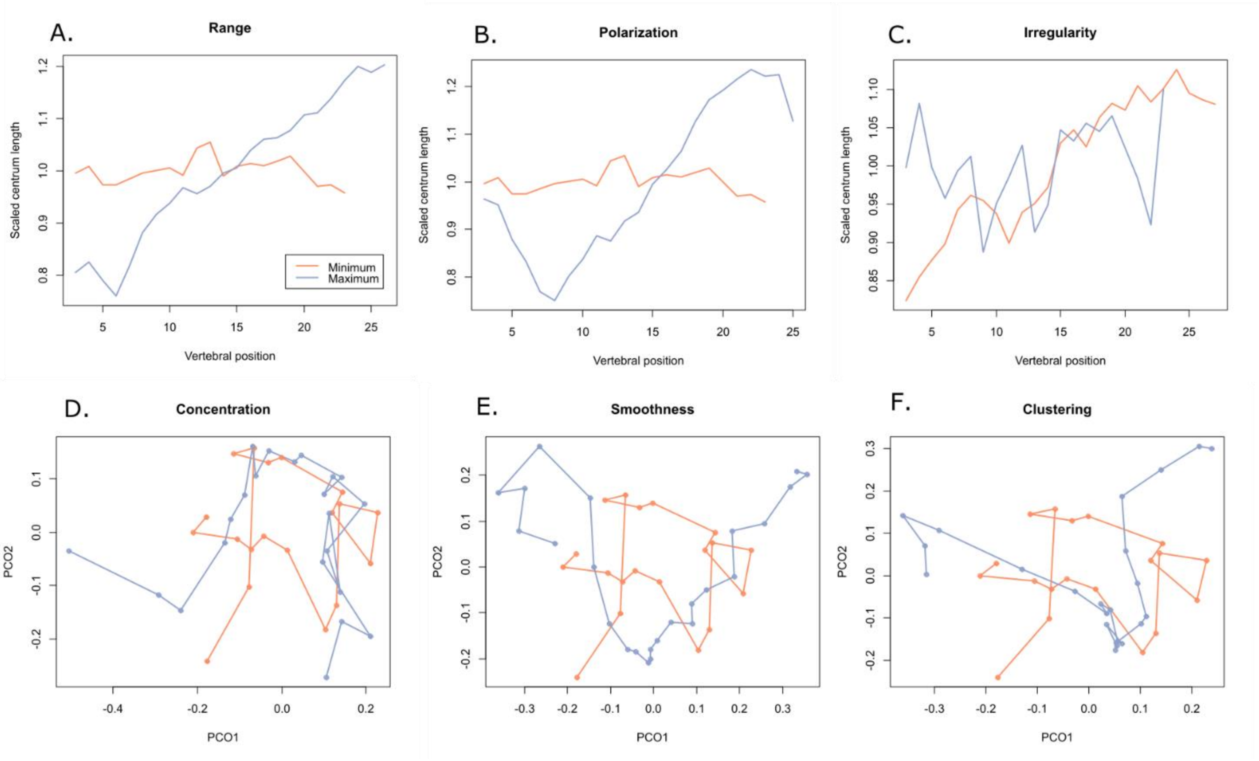

Supplementary Figure 1. Graphs illustrating the maximum and minimum of each of the complexity and organization metrics. Using scaled centrum length as an example, A. range reflects maximum spread of the vertebrae, B. polarization reflects their average spread, while C. irregularity reflects the variation between adjacent vertebrae. Principal coordinates analysis was used to demonstrate the relative distribution of vertebrae captured in the organization metrics. D. Concentration represents the clumping of the vertebrae near the mean shape versus their dispersal at the extremes, E. smoothness reflects the gradient of morphology along the vertebral column, and F. clustering represents the non-randomness of their distribution

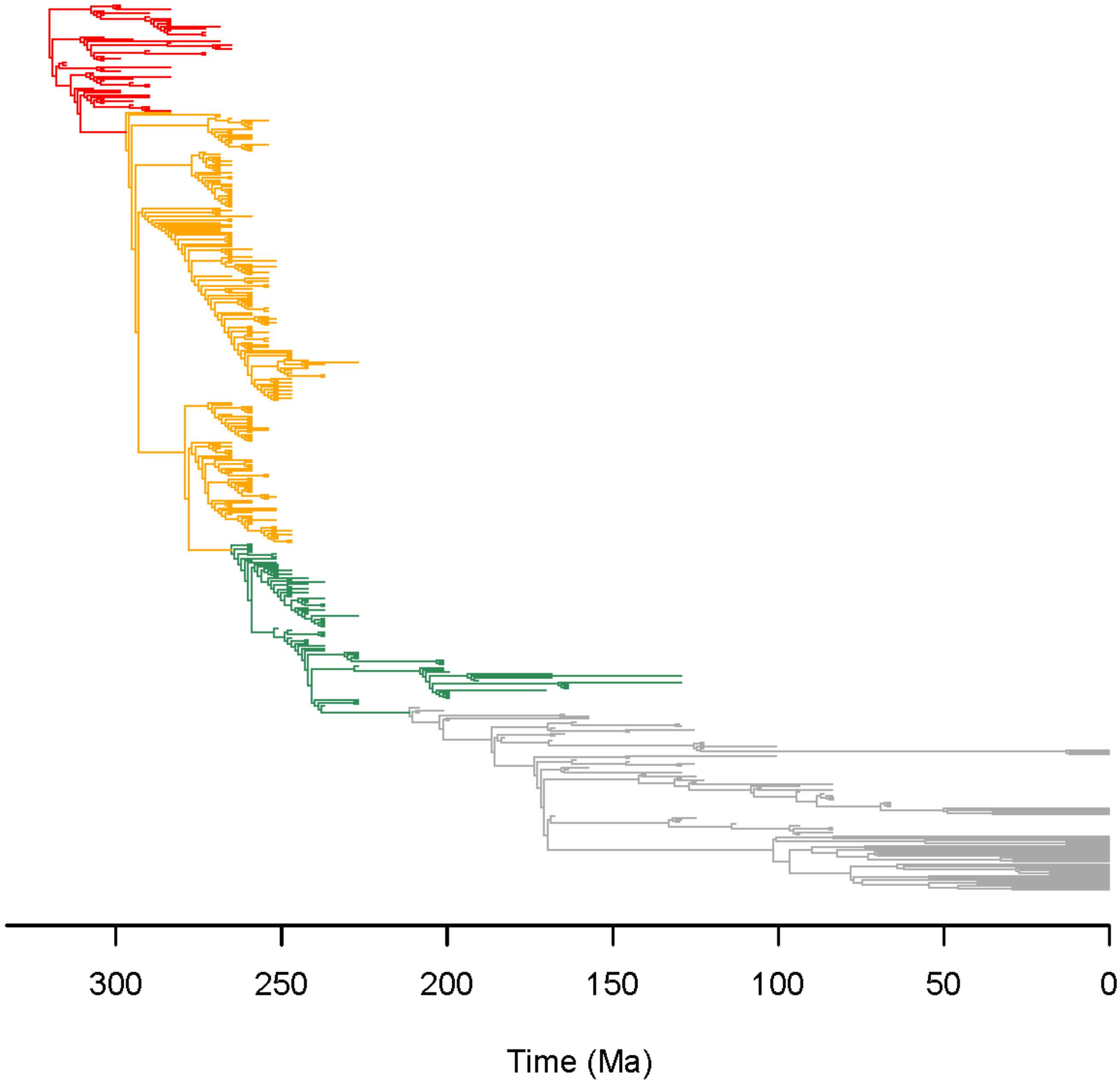

Supplementary Figure 2. 'Master' phylogeny including the 444 tips used for Monte Carlo simulations. The master tree was subsampled during simulations to estimate effects of sampling on model selection. Red: 'pelycosaurs', n=45; orange: non-cynodont therapsids, n=226; green: non-mammalian cynodonts, n=82; grey: mammaliaforms, n=91. Phylogeny is available on dryad (https://doi.org/10.5061/dryad.5mkkwh71h).

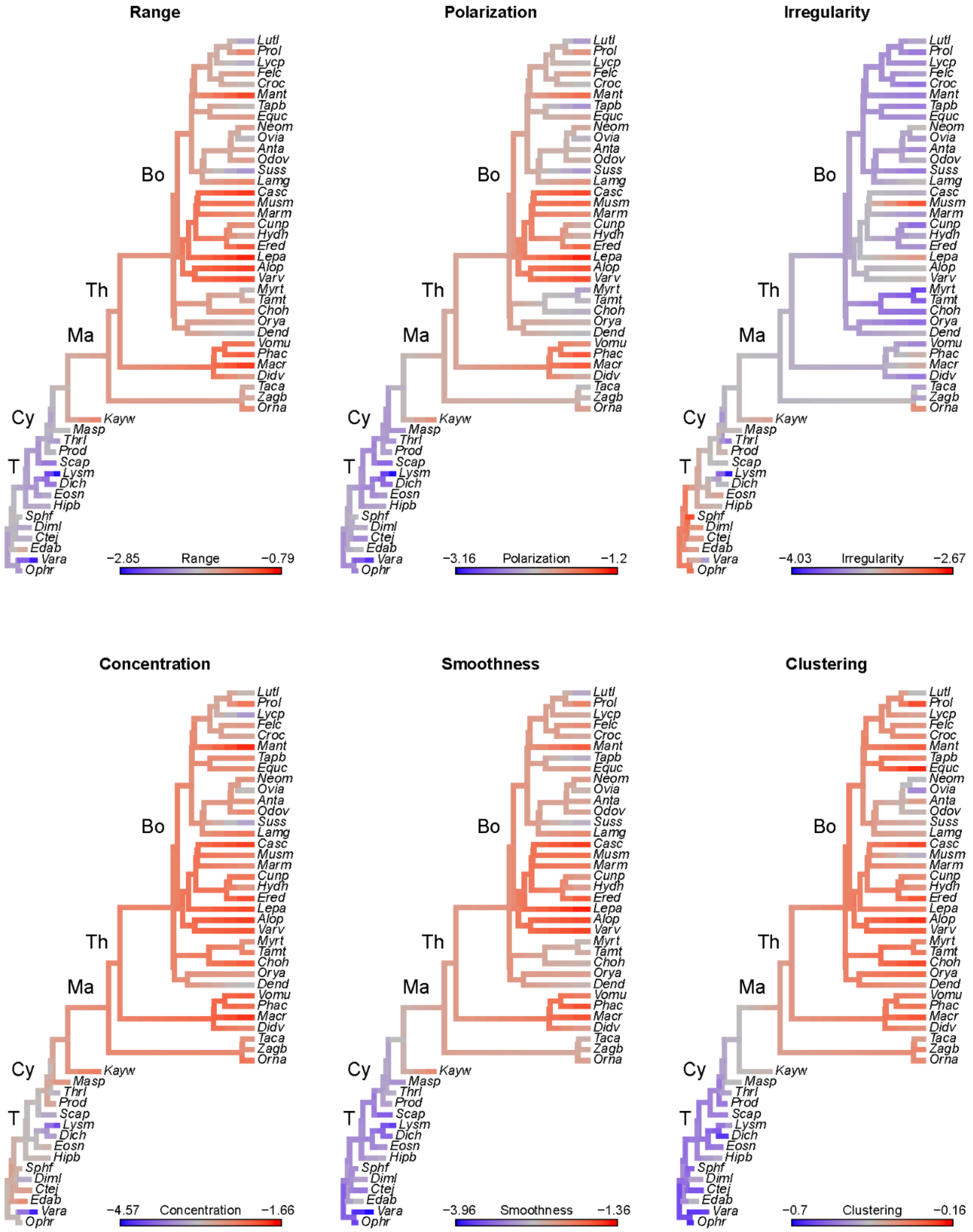

Supplementary Figure 3. Variation in vertebral complexity (top) and organization (bottom) metrics across synapsids for centrum-only dataset, optimized onto the phylogeny using maximum likelihood.

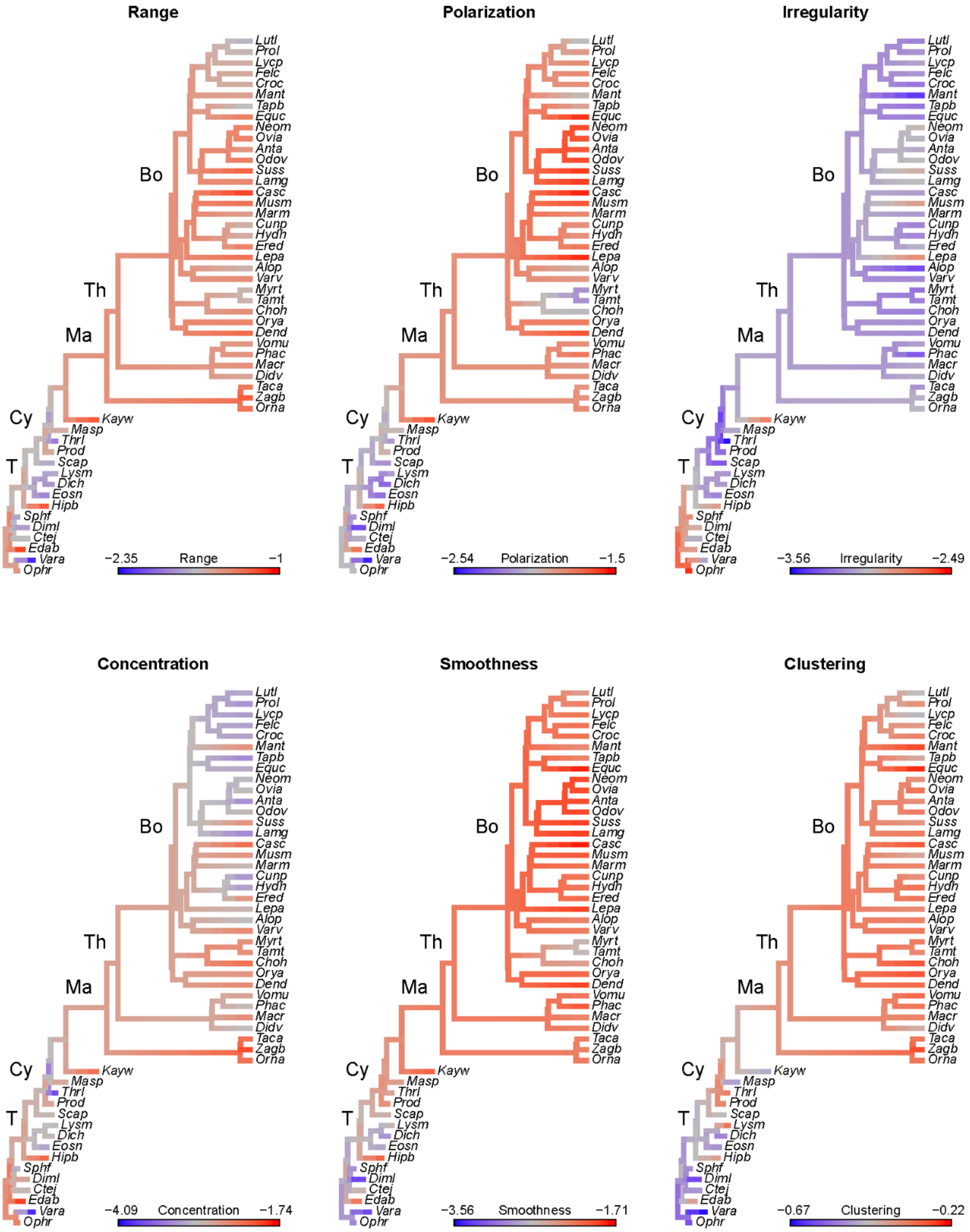

Supplementary Figure 4. Variation in vertebral complexity (top) and organization (bottom) metrics across synapsids for arch-only dataset, optimized onto the phylogeny using maximum likelihood.

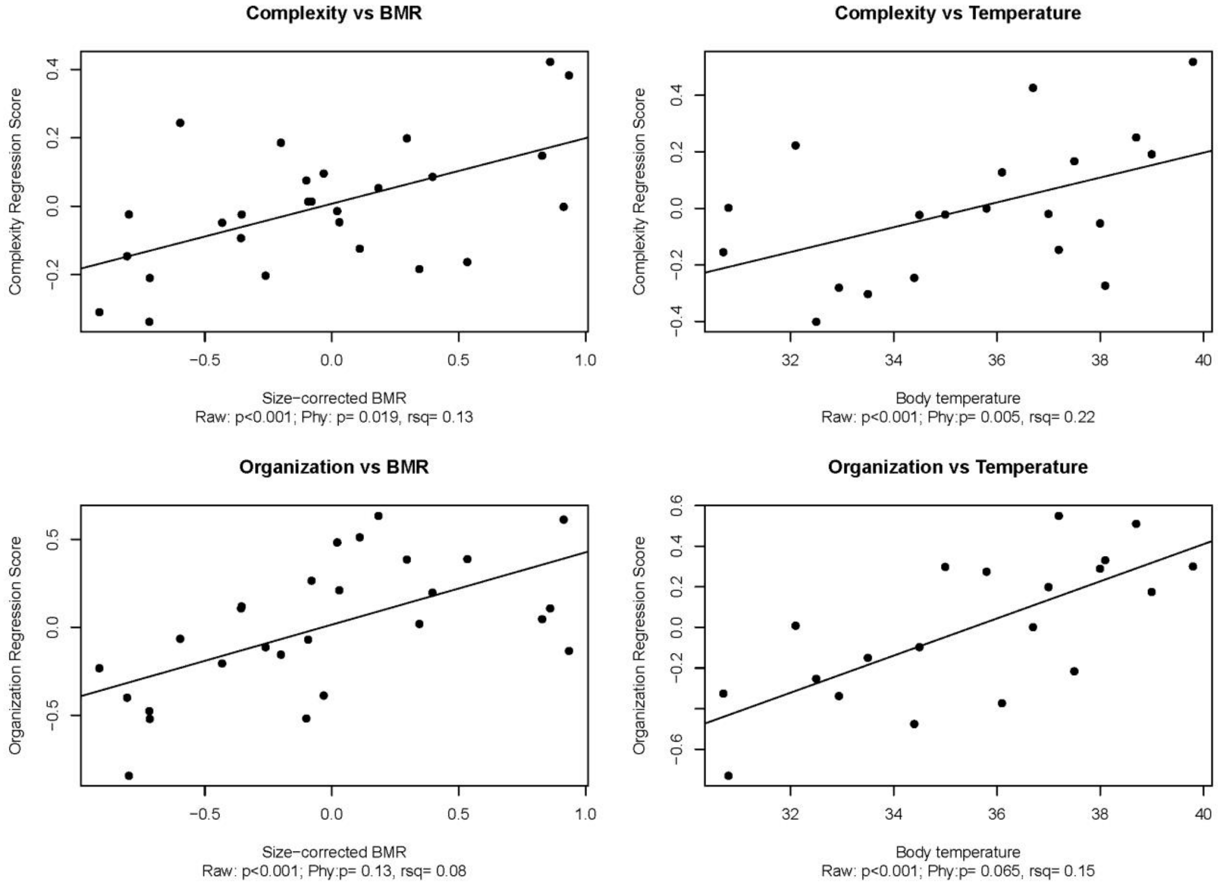

Supplementary Figure 5. Phylogenetically-corrected regressions of complexity and organization metrics with basal metabolic rates and body temperature for extant mammals. Graphs illustrate the regression scores, the multivariate component of variation most highly correlated with the independent variable. Raw (multivariate regression) and phylogenetically-corrected (PGLS) p-values and r-squared values are shown below.

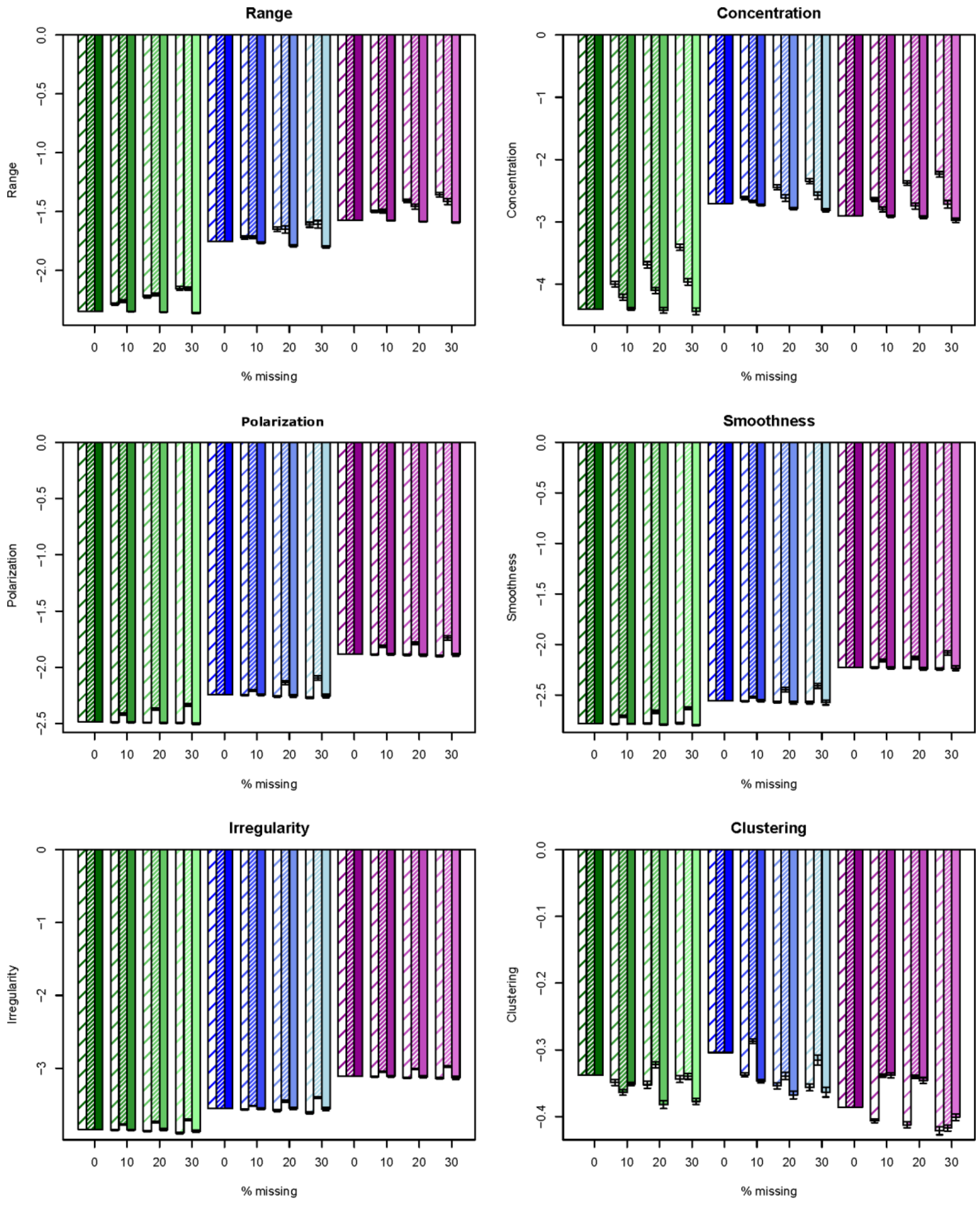

Supplementary Figure 6. Sensitivity of complexity and organization metrics to missing data. Green: Thrinaxodon liorhinus*; Blue:* Tachyglossus aculeatus*; Purple:* Mus musculus*. Light-dash fill: element-wise removal; Medium-dash fill: variable removal; Solid fill: vertebra removal. Source data are provided as a Source Data file.*

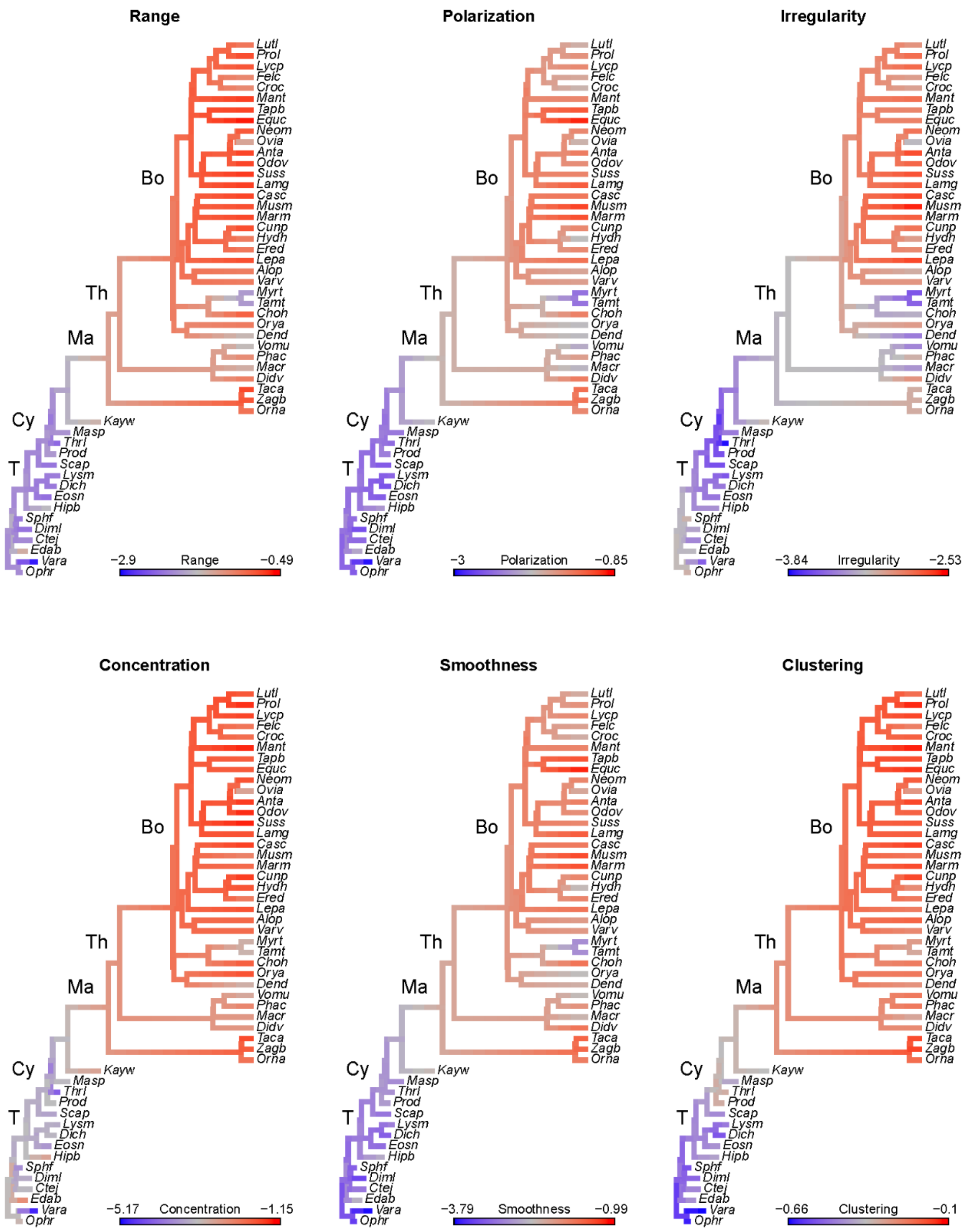

Supplementary Figure 7. Variation in vertebral complexity (top) and organization (bottom) metrics across synapsids with serial absent structures coded as AbsSmall.

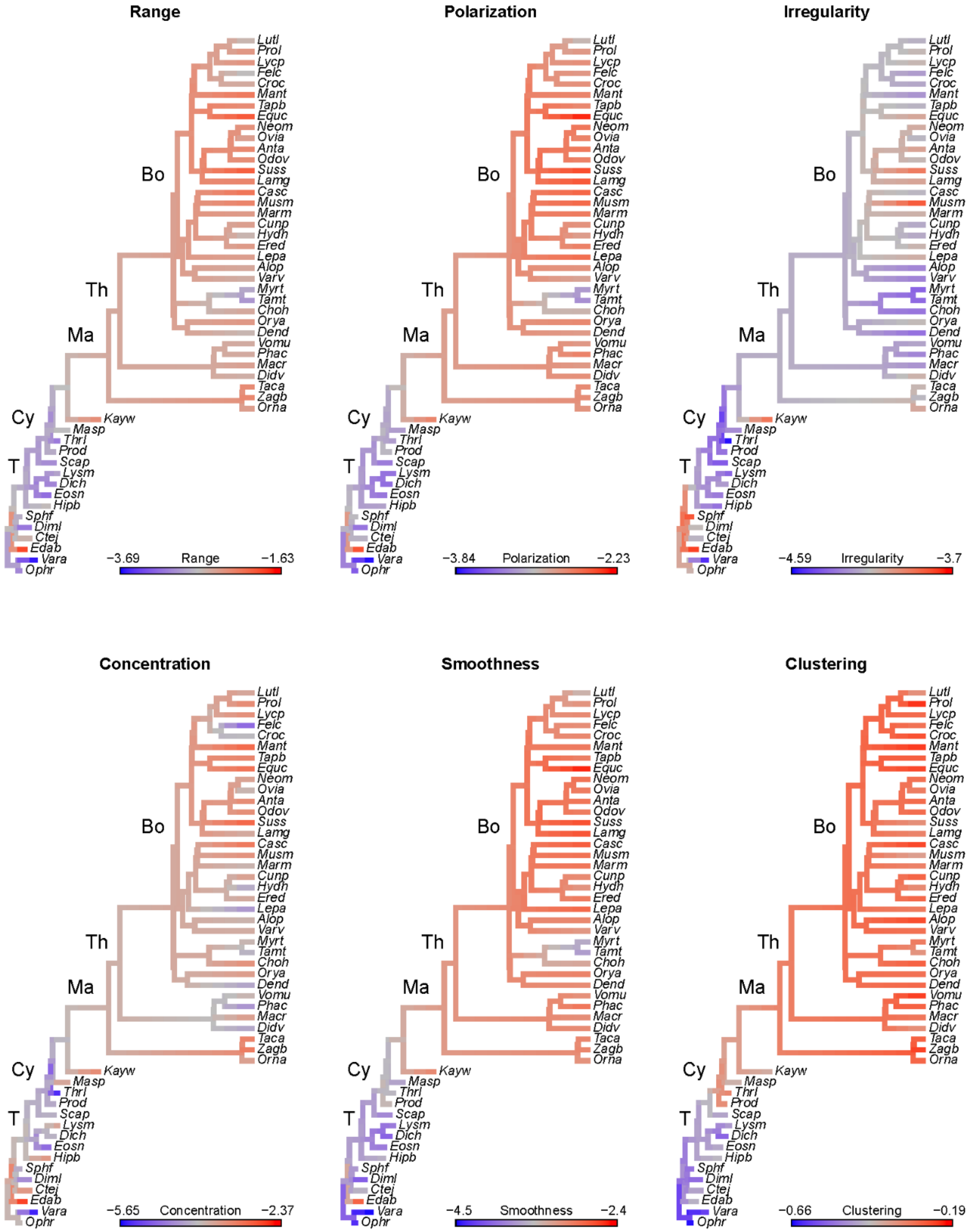

Supplementary Figure 8. Variation in vertebral complexity (top) and organization (bottom) metrics across synapsids based on serial absent structures coded as AbsOne.

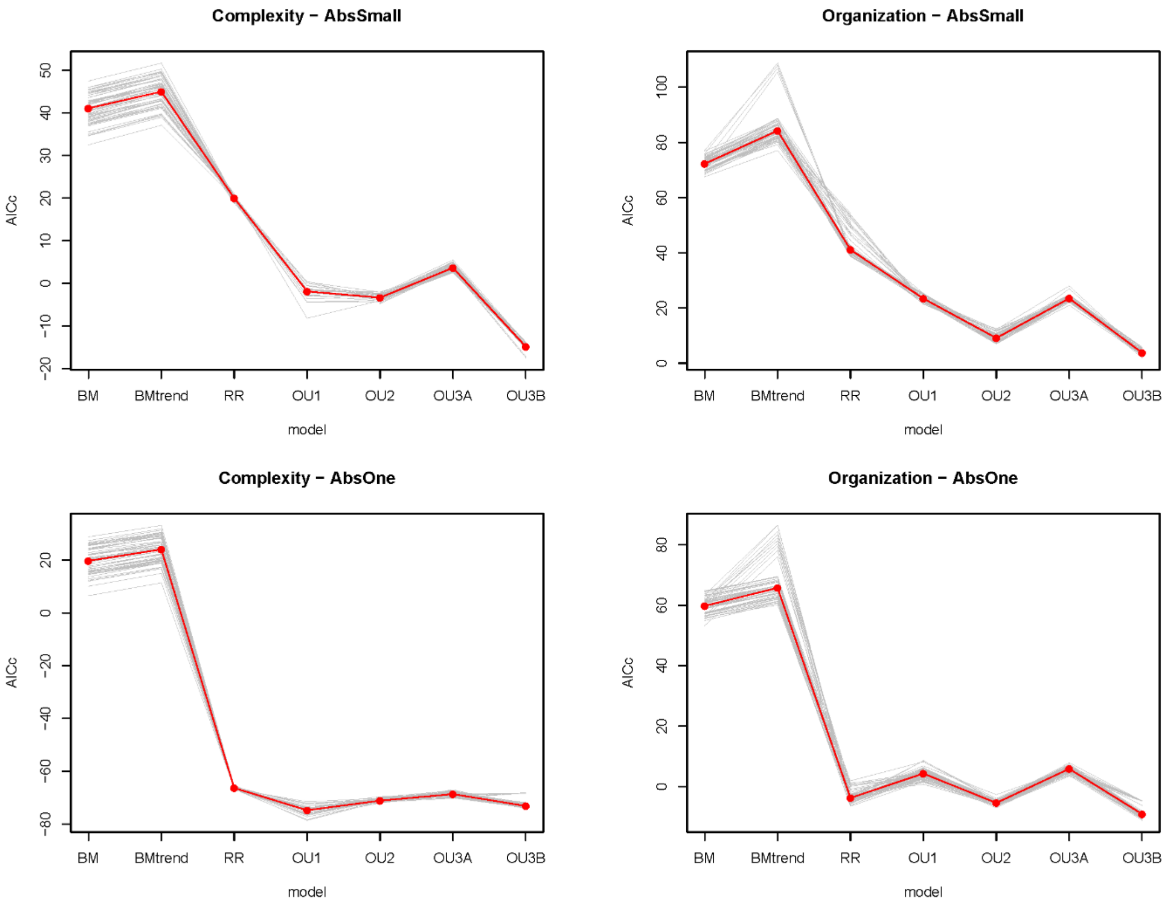

Supplementary Figure 9. Comparison of model performance based on AICc across 60 trees for complexity (left) and organization (right) including serial absent structures as AbsSmall (top) and AbsOne (bottom). For model definitions see Table 1. Source data are provided as a Source Data file.

Supplementary Tables

Supplementary Table 1. Median optima values (θ) for each model from the whole vertebra dataset. For parameter definitions see Table 1. Ra: range; Po: polarization; Ir: irregularity; Co: concentration; Sm: smoothness; Cl: clustering; UCI: upper confidence interval; LCI: lower confidence interval. Confidence intervals generated from jackknifing.

		Complexity									Organization								
		Ra	UCI	LCI	Po	UCI	LCI	Ir	UCI	LCI	Co	UCI	LCI	Sm	UCI	LCI	Cl	UCI	LCI
**BM**	theta	-2.01	-2.11	-1.98	-2.44	-2.48	-2.42	-3.19	-3.26	-3.17	-3.14	-3.30	-3.07	-3.16	-3.19	-3.11	-0.56	-0.57	-0.54
**Trend**	theta	-2.02	-2.14	-1.96	-2.45	-2.49	-2.41	-3.15	-3.21	-3.12	-3.08	-3.16	-3.05	-3.17	-3.26	-3.02	-0.57	-0.59	-0.54
**RR**	theta	-2.05	-2.11	-1.78	-2.42	-2.48	-2.21	-3.45	-3.50	-3.27	-3.28	-3.44	-2.70	-2.91	-3.07	-2.69	-0.47	-0.53	-0.42
**OU1**	root	-3.11	-6.32	-2.35	-2.07	-2.68	0.02	-1.89	-2.30	0.64	-3.92	-5.32	-1.53	-3.52	-3.75	-3.32	-0.79	-1.00	-0.61
	theta	-1.80	-1.82	-1.80	-2.13	-2.14	-2.12	-3.48	-3.49	-3.47	-3.16	-3.18	-3.13	-2.40	-2.42	-2.39	-0.32	-0.32	-0.32
**OU2**	NMS	-2.14	-2.16	-2.06	-2.49	-2.53	-2.43	-3.47	-3.48	-3.42	-3.28	-3.46	-3.20	-3.00	-3.03	-2.88	-0.48	-0.49	-0.47
	mammal	-1.75	-1.76	-1.74	-2.07	-2.08	-2.06	-3.47	-3.48	-3.46	-3.12	-3.15	-3.10	-2.36	-2.37	-2.35	-0.31	-0.31	-0.31
**OU3A**	NTS	-2.16	-2.25	-2.05	-2.55	-2.65	-2.49	-3.30	-3.39	-3.26	-3.12	-3.59	-3.00	-3.03	-3.33	-2.94	-0.52	-0.56	-0.51
	therapsid	-1.96	-2.04	-1.86	-2.36	-2.42	-2.25	-3.48	-3.51	-3.40	-3.19	-3.25	-3.07	-2.77	-2.84	-2.65	-0.42	-0.43	-0.40
	theria	-1.76	-1.77	-1.75	-2.07	-2.08	-2.05	-3.48	-3.49	-3.47	-3.16	-3.18	-3.13	-2.36	-2.37	-2.35	-0.31	-0.31	-0.31
**OU3B**	NCS	-2.16	-2.19	-2.13	-2.55	-2.57	-2.52	-3.39	-3.43	-3.37	-3.25	-3.38	-3.13	-3.10	-3.16	-3.01	-0.53	-0.53	-0.50
	cynodont	-1.75	-1.79	-1.74	-2.16	-2.20	-2.14	-3.48	-3.51	-3.46	-2.92	-3.01	-2.89	-2.47	-2.53	-2.45	-0.31	-0.33	-0.31
	boreotheria	-1.77	-1.79	-1.76	-2.04	-2.06	-2.03	-3.46	-3.48	-3.46	-3.27	-3.31	-3.26	-2.31	-2.34	-2.31	-0.31	-0.32	-0.31

Supplementary Table 2. Median rate matrices for each model from the whole vertebra dataset.

		Complexity			Organization			
		Range	Polarization	Irreg.		Conc.	Smooth.	Cluster
**BM**	Range	0.0019	0.0011	0.0006	Conc.	0.0118	0.0020	0.0003
	Polarization	0.0011	0.0009	0.0005	Smooth.	0.0020	0.0019	0.0003
	Irregularity	0.0006	0.0005	0.0011	Cluster	0.0003	0.0003	0.0001
**BMtrend**	Range	0.0019	0.0011	0.0006	Conc.	0.0118	0.0020	0.0003
	Polarization	0.0011	0.0009	0.0005	Smooth.	0.0020	0.0018	0.0003
	Irregularity	0.0006	0.0005	0.0011	Cluster	0.0003	0.0003	0.0001
**RR**	Range	0.0090	0.0020	0.0043	Conc.	0.0402	0.0023	0.0030
	Polarization	0.0020	0.0019	0.0026	Smooth.	0.0023	0.0050	0.0013
	Irregularity	0.0043	0.0026	0.0090	Cluster	0.0030	0.0013	0.0005
**OU1**	Range	0.0071	0.0016	-0.0004	Conc.	0.0762	0.0057	0.0043
	Polarization	0.0016	0.0013	0.0009	Smooth.	0.0057	0.0024	0.0009
	Irregularity	-0.0004	0.0009	0.0032	Cluster	0.0043	0.0009	0.0004
**OU2**	Range	0.0111	0.0024	0.0010	Conc.	0.0742	0.0091	0.0053
	Polarization	0.0024	0.0014	0.0012	Smooth.	0.0091	0.0042	0.0016
	Irregularity	0.0010	0.0012	0.0032	Cluster	0.0053	0.0016	0.0006
**OU3A**	Range	0.0101	0.0007	-0.0002	Conc.	0.0699	0.0017	0.0038
	Polarization	0.0007	0.0004	0.0004	Smooth.	0.0017	0.0030	0.0009
	Irregularity	-0.0002	0.0004	0.0115	Cluster	0.0038	0.0009	0.0004
**OU3B**	Range	0.0206	-0.0025	-0.0005	Conc.	0.1067	-0.0233	-0.0127
	Polarization	-0.0025	0.0039	-0.0072	Smooth.	-0.0233	0.0055	0.0029
	Irregularity	-0.0005	-0.0072	0.0329	Cluster	-0.0127	0.0029	0.0016

Supplementary Table 3. Phylogenetic half-life of Ornstein-Uhlenbeck models from the whole-vertebra dataset. Phylogenetic half-life is inversely proportional to alpha, the strength of the pull toward the optimum. Values that are large relative to tree height indicate weak pull and suggest Brownian motion-type dynamics. Phylogenetic half-life calculated from eigenvalues of alpha matrices in mvMORPH.

	**Complexity**			**Organization**		
	Range	Polarization	Irregularity	Conc.	Smooth.	Cluster
**OU1**	3.71	11.05	34.80	4.82	20.60	33.99
**OU2**	3.15	12.98	19.51	5.06	13.03	27.25
**OU3A**	1.22	3.95	23.73	4.86	15.53	18.97
**OU3B**	0.38	2.17	16.03	1.37	3.68	12.33

Supplementary Table 4. ANOVA of between-group differences of tip values for OU3B model. Pairwise comparisons from TukeyHSD test. NCS: non-cynodont synapsid; Cyn: non-boreoeutherian cynodont; Bor: boreoeutherian.

	Range	Polarization	Irregularity	Concentration	Smoothness	Clustering
**F-value**	**9.12**	**23.01**	0.93	2.29	**33.39**	**54.72**
**P-value**	**<0.001**	**<0.001**	0.402	0.113	**<0.001**	**<0.001**
**NCS-Cyn**	**-0.34**	**-0.33**	-	-	**-0.58**	**-0.21**
**NCS-Cyn P-val**	**0.003**	**0.003**	-	-	**<0.001**	**<0.001**
**Cyn-Bor**	-0.03	**-0.17**	-	-	**-0.22**	0.00
**Cyn-Bor P-val**	0.880	**0.032**	-	-	**0.037**	0.985

Supplementary Table 5. Relative fit and rank order of evolutionary models including the cynodont-only and boreoeutherian-only shifts. AICc: Akaike information criterion; diff: AICc difference; wi: weighting; AICw: Akaike weight. Median and range values drawn from analysis across 60 phylogenetic trees. Grey shading: best model used for H1 in hypothesis test simulations.

	Rank	AIC	diff	wi	AICw
**Complexity**					
**OU3B**	1	-105.6	0.0	1.00	0.88
**OU1**	2	-100.0	5.6	0.06	0.05
**OU3A**	3	-99.8	5.8	0.06	0.05
**cyn**	4	-96.8	8.8	0.01	0.01
**boreo**	5	-95.5	10.0	0.01	0.01
**OU2**	6	-93.4	12.2	0.00	0.00
**RR**	7	-81.8	23.7	0.00	0.00
**BM**	8	-25.5	80.0	0.00	0.00
**BMtrend**	9	-20.7	84.8	0.00	0.00
**Organization**					
**OU3B**	1	-56.5	0.0	1.00	0.93
**cyn**	2	-51.2	5.4	0.07	0.06
**RR**	3	-41.5	15.1	0.00	0.00
**OU2**	4	-41.5	15.1	0.00	0.00
**OU1**	5	-39.4	17.1	0.00	0.00
**OU3A**	6	-37.2	19.4	0.00	0.00
**boreo**	7	-31.5	25.0	0.00	0.00
**BM**	8	20.5	77.0	0.00	0.00
**BMtrend**	9	26.0	82.6	0.00	0.00

Supplementary Table 6. Hypothesis tests of best models (H1) against various null models (H0) including cynodont-only and boreoeutherian-only model. Log likelihood ratio tests (LR) based on Monte Carlo simulations with taxonomic down-sampling and trees drawn at random from 60 phylogenies. LR are jack-knifed median values with 95% confidence intervals (LCI, UCI). Th: LR threshold required to reject the null hypothesis based on simulations.

H0	H1	LnLik	LCI	UCI	Th	P-val
**Complexity**						
**BM**	OU3B	109.8	95.9	114.4	23.3	0.00
**BMtrend**		107.5	93.1	112.7	20.0	0.00
**RR**		23.7	5.9	81.6	-2.0	0.00
**OU1**		12.1	5.7	15.4	1.7	0.00
**OU2**		20.2	12.0	21.8	9.0	0.00
**OU3A**		5.8	2.2	16.9	-1.7	0.00
**Cyn**		16.8	11.3	17.1	9.2	0.00
**Boreo**		18.2	15.4	20.5	9.2	0.00
**Organization**						
**BM**	OU3B	107.0	92.6	114.9	24.1	0.00
**BMtrend**		105.6	89.2	129.1	58.4	0.00
**RR**		15.4	8.7	88.5	-1.5	0.00
**OU1**		25.2	16.0	30.4	5.7	0.00
**OU2**		23.0	14.1	30.1	5.8	0.00
**OU3A**		19.3	16.8	28.7	0.0	0.00
**Cyn**		13.4	10.4	14.8	8.9	0.01
**Boreo**		32.9	27.7	36.5	9.8	0.00

Supplementary Table 7. Taxonomic sample. Bold indicates fossil taxa.

Species	Abbreviation	Sp. No
Ctenorhachis jacksoni	**Ctej**	***USNM 437710***
Dicynodon huenei	**Dich**	***NHMUK PV R37005***
Dimetrodon limbatus	**Diml**	***AMNH FARB 4008***
Edaphosaurus boanerges	**Edab**	***DMNH 2011-04-01***
Eosimops newtoni	**Eosn**	***BP/1/6674***
Hipposaurus boonstrai	**Hipb**	***SAM-PK-8950***
Kayentatherium wellesi	**Kayw**	***MCZ 8812***
Lystrosaurus murrayi	**Lysm**	***UMZC T763***
Massetognathus pascuali	**Masp**	***MCZ 3691***
Ophiacodon retroversus	**Ophr**	***FMNH UC 458***
Procynosuchus delaharpaea	**Prod**	***TSK 34***
Scalaposaurus punctatus	**Scap**	***UMZC T837***
Sphenacodon ferox	**Sphf**	***YPM 818***
Thrinaxodon liorhinus	**Thrl**	***BP/1/7199***
Varanosaurus acutirostris	**Vara**	***AMNH FARB 4174***
Alouatta palliata	Alop	MCZ 47267
Antilocapra americana	Anta	MCZ 1773
Castor canadensis	Casc	MCZ 64159
Choloepus hoffmani	Choh	MCZ 12348
Crocuta crocuta	Croc	MCZ 20968
Cuniculus paca	Cunp	MCZ 829
Dendrohyrax dorsalis	Dend	MCZ 6069
Didelphis virginiana	Didv	MCZ 62096
Equus caballus	Equc	MCZ 14915
Erethizon dorsatum	Ered	MCZ 965
Felis catus	Felc	MCZ 68415
Hydrochoerus hydrochaeris	Hydh	MCZ 6013
Lama glama	Lamg	MCZ BOM1881
Lepus americanus	Lepa	MCZ 852
Lutra lutra	Lutl	UMZC K2768
Lycaon pictus	Lycp	MCZ 13233
Macropus robustus	Macr	MCZ 63609
Manis temminckii	Mant	MCZ 34184
Marmota monax	Marm	MCZ 377
Mus musculus	Musm	MCZ 59560
Myrmecophaga tridactyla	Myrt	MCZ 20969
Neotragus moschatus	Neom	MCZ 53804
Odocoileus virginianus	Odov	MCZ 46590
Ornithorhynchus anatinus	Orna	USNM 221110
Orycteropus afer	Orya	MCZ 20970

Ovis aries	Ovia	MCZ 6338
Phascolarctos cinereus	Phac	MCZ 58136
Procyon lotor	Prol	MCZ 7101
Sus scrofa	Suss	MCZ 6246
Tachyglossus aculeatus	Taca	MCZ 63621
Tamandua tetradactyla	Tamt	MCZ 20965
Tapirus bairdii	Tapb	MCZ 1076
Varecia veregata	Varv	MCZ 18740
Vombatus ursinus	Vomu	MCZ 24974
Zaglossus bruijnii	Zagb	MCZ 12414

MCZ: Museum of Comparative Zoology; UMZC: University Museum of Zoology Cambridge; FMNH: Field Museum of Natural History; AMNH: American Museum of Natural History; YPM: Yale Peabody Museum; NHMUK: Natural History Museum, London; BP: Evolutionary Studies Institute; SAM: Iziko Museums of South Africa; DMNH: Dallas Museum of Natural History.

Supplementary Table 8: Morphometric measurements and vertebra module subset. More information available in Jones et al.[56]

Module	Measurement	Description
<u>**Centrum**</u>		
	**CL**	Centrum Length
	**CHPost**	Centrum height (posterior)
	**CWPost**	Centrum width (posterior)
	**CHant**	Centrum height (anterior)
	**CWant**	Centrum width (anterior)
<u>**Neural Arch**</u>		
	**ArchH**	Arch height
	**ArchW**	Arch width
	**MidLW**	Mid-lamina width
	**NSL**	Neural spine length
	**NSH**	Neural spine height
	**TotH**	Total height
	**TotW**	Total width
	**PreZw**	Pre-zygapophysis width
	**InterZL**	Inter-zygapophyseal length
	**TPL**	Transverse process/diapophysis length

Supplementary Table 9. Relative fit and rank order of evolutionary models including serial absent structures. AICc: Akaike information criterion; diff: AICc difference; wi: weighting; AICw: Akaike weight. Median and range values drawn from analysis across 60 phylogenetic trees.

	AbsSmall						AbsOne				
	Rank	AICc	diff	wi	AICw		Rank	AICc	diff	wi	AICw
**Complexity**						**Complexity**					
**OU3B**	1	-14.9	0.0	1.00	1.00	**OU1**	1	-74.8	0.0	1.00	0.60
**OU2**	2	-3.3	11.5	0.00	0.00	**OU3B**	2	-73.2	1.6	0.45	0.27
**OU1**	3	-1.9	13.0	0.00	0.00	**OU2**	3	-71.1	3.7	0.16	0.10
**OU3A**	4	3.7	18.5	0.00	0.00	**OU3A**	4	-68.7	6.1	0.05	0.03
**RR**	5	20.0	34.9	0.00	0.00	**RR**	5	-66.3	8.4	0.01	0.01
**BM**	6	41.1	55.9	0.00	0.00	**BM**	6	19.7	94.5	0.00	0.00
**BMtrend**	7	45.0	59.9	0.00	0.00	**BMtrend**	7	24.0	98.8	0.00	0.00
**Organization**						**Organization**					
**OU3B**	1	3.8	0.0	1.00	0.94	**OU3B**	1	-9.1	0.0	1.00	0.81
**OU2**	2	9.1	5.3	0.07	0.06	**OU2**	2	-5.4	3.7	0.16	0.13
**OU1**	3	23.4	19.7	0.00	0.00	**RR**	3	-3.8	5.4	0.07	0.06
**OU3A**	4	23.5	19.7	0.00	0.00	**OU1**	4	4.3	13.4	0.00	0.00
**RR**	5	41.1	37.3	0.00	0.00	**OU3A**	5	5.9	15.0	0.00	0.00
**BM**	6	72.3	68.5	0.00	0.00	**BM**	6	59.8	68.9	0.00	0.00
**BMtrend**	7	84.2	80.5	0.00	0.00	**BMtrend**	7	65.8	74.9	0.00	0.00

Supplementary Table 10. Hypothesis tests of OU3B (H1) against various null models (H0) for serial absent structures. Log likelihood ratio tests (LR) based on Monte Carlo simulations with taxonomic down-sampling and trees drawn at random from 60 phylogenies. LR are median values with 95% confidence intervals (CImin, CImax), generated from jack-knifing. Thresh: LR threshold required to reject the null hypothesis based on simulations. Bold: Non-significant based on median; Grey shading: Non-significant based on lower confidence interval.

	**AbsSmall**					**AbsOne**				
**h0**	LR	Cimin	Cimax	Thresh	P-value	LR	Cimin	Cimax	Thresh	P-value
	**Complexity**					**Complexity**				
**BM**	77.53	68.77	85.36	23.49	0.000	123.11	109.35	132.81	22.96	0.000
**BMtrend**	73.80	63.01	81.51	18.96	0.000	119.63	101.27	126.73	18.72	0.000
**RR**	42.71	6.28	103.78	-2.69	0.000	11.43	-6.57	82.37	-11.54	0.000
**OU1**	18.32	12.29	26.98	5.95	0.007	*5.05*	*-5.08*	*10.43*	*-0.27*	*0.015*
**OU2**	23.75	14.89	30.12	9.09	0.002	*9.79*	*3.05*	*12.95*	*6.33*	*0.020*
**OU3A**	21.51	12.70	33.94	2.66	0.011	*5.92*	*0.20*	*11.66*	*1.25*	*0.016*
	**Organization**					**Organization**				
**BM**	97.52	88.71	102.20	23.65	0.000	101.61	86.91	107.72	24.21	0.000
**BMtrend**	100.52	90.40	108.67	59.72	0.005	99.84	85.82	113.18	54.66	0.000
**RR**	59.73	32.63	118.33	3.19	0.000	27.32	-2.26	76.66	-11.31	0.000
**OU1**	26.83	19.73	33.91	4.80	0.000	24.63	14.85	29.28	4.63	0.000
**OU2**	19.92	11.04	25.96	1.52	0.000	17.15	6.43	19.81	4.06	0.000
**OU3A**	21.77	16.84	31.68	0.81	0.000	20.22	11.91	24.11	1.89	0.000

Supplementary Table 11. Regression coefficients for raw and phylogenetically-corrected regressions of complexity and organization on BMR and body temperature.

X	Analysis	Parameter	Range	Polarization	Irregularity	Concentration	Smoothness	Clustering
**BMR**	Raw	Intercept	-1.753	-2.080	-3.459	-3.097	-2.377	-0.317
**BMR**	Raw	Slope	0.003	0.168	0.137	-0.385	0.182	-0.033
**BMR**	Corrected	Intercept	-1.723	-2.068	-3.403	-3.014	-2.381	-0.323
**BMR**	Corrected	Slope	-0.025	0.094	0.209	-0.273	0.052	-0.053
**Temperature**	Raw	Intercept	-1.932	-3.598	-4.259	-0.252	-4.175	-0.156
**Temperature**	Raw	Slope	0.005	0.042	0.023	-0.077	0.049	-0.004
**Temperature**	Corrected	Intercept	-1.928	-4.072	-5.901	0.639	-4.201	-0.145
**Temperature**	Corrected	Slope	0.006	0.057	0.071	-0.103	0.052	-0.005

Supplementary References

1. Brocklehurst N, Reisz RR, Fernandez V, Fröbisch J. A re-description of '*Mycterosaurus' smithae*, an Early Permian eothyridid, and its impact on the phylogeny of pelycosaurian-grade synapsids. *PLoS ONE* **11**, e0156810 (2016).

2. Modesto SP. The skull of the herbivorous synapsid *Edaphosaurus boanerges* from the Lower Permian of Texas. *Palaeontology* **38**, 213-239 (1995).

3. Brink KS, Maddin HC, Evans DC, Reisz RR. Re-evaluation of the historic Canadian fossil *Bathygnathus borealis* from the Early Permian of Prince Edward Island. *Can J Earth Sci* **52**, 1109-1120 (2015).

4. Sidor CA, Hopson JA. Ghost lineages and "mammalness": assessing the temporal pattern of character acquisition in the Synapsida. *Paleobiology* **24**, 254-273 (1998).

5. Day MO, Smith RM, Benoit J, Fernandez V, Rubidge BS. A new species of burnetiid (Therapsida, Burnetiamorpha) from the early Wuchiapingian of South Africa and implications for the evolutionary ecology of the family Burnetiidae. *Papers in Palaeontology* **4**, 453-475 (2018).

6. Kammerer CF. Systematics of the Anteosauria (Therapsida: Dinocephalia). *J Syst Palaeontol* **9**, 261-304 (2011).

7. Cisneros JC, Abdala F, Atayman-Güven S, Rubidge BS, Şengör AC, Schultz CL. Carnivorous dinocephalian from the Middle Permian of Brazil and tetrapod dispersal in Pangaea. *Proceedings of the National Academy of Sciences* **109**, 1584-1588 (2012).

8. Rubidge BS, Heever JA. Morphology and systematic position of the dinocephalian *Styracocephalus platyrhynchus*. *Lethaia* **30**, 157-168 (1997).

9. Atayman S, Rubidge BS, Abdala F. Taxonomic re-evaluation of tapinocephalid dinocephalians. *Palaeontol Afr* **44**, 88-90 (2009).

10. Güven S, Rubidge BS, Abdala F. Cranial morphology and taxonomy of South African Tapinocephalidae (Therapsida, Dinocephalia): the case of *Avenantia* and *Riebeeckosaurus*. *Palaeontol Afr* **48**, 24-33 (2013).

11. Angielczyk KD, Kammerer CF. The cranial morphology, phylogenetic position and biogeography of the upper Permian dicynodont *Compsodon helmoedi* van Hoepen (Therapsida, Anomodontia). *Papers in Palaeontology* **3**, 513-545 (2017).

12. Olroyd SL, Sidor CA, Angielczyk KD. New materials of the enigmatic dicynodont *Abajudon kaayai* (Therapsida, Anomodontia) from the lower Madumabisa Mudstone Formation, middle Permian of Zambia. *J Vert Paleontol* **37**, e1403442 (2017).

13. Kammerer CF, Masyutin V. Gorgonopsian therapsids (*Nochnitsa* gen. nov. and *Viatkogorgon*) from the Permian Kotelnich locality of Russia. *PeerJ* **6**, e4954 (2018).

14. Kammerer CF, Masyutin V. A new therocephalian (*Gorynychus masyutinae* gen. et sp. nov.) from the Permian Kotelnich locality, Kirov Region, Russia. *PeerJ* **6**, e4933 (2018).

15. Huttenlocker AK, Smith RHM. New whaitsioids (Therapsida: Therocephalia) from the Teekloof Formation of South Africa and therocephalian diversity during the end-Guadalupian extinction. *PeerJ* **5**, e3868 (2017).

16. Van den Brandt MJ, Abdala F. Cranial morphology and phylogenetic analysis *Cynosurus suppostus* (Therapsida, Cynodontia) form the upper Permian of the Karoo Basin, South Africa. *Palaeontol Afr* **52**, 201-221 (2018).

17. Ray S. A new Late Triassic traversodontid cynodont (Therapsida, Eucynodontia) from India. *J Vert Paleontol* **35**, e930472 (2015).

18. Pavanatto A, Pretto FA, Kerber L, Muller R, Da-Rosa AAS, Dias-Da-Silva S. A new Upper Triassic cynodont-bearing fossiliferous site from southern Brazil, with taphonomic remarks and description of a new traversodontid taxon. *J South Am Earth Sci* **88**, 179-196 (2018).

19. Ruta M, Botha-Brink J, Mitchell SA, Benton MJ. The radiation of cynodonts and the ground plan of mammalian morphological diversity. *Proceedings Biological sciences* **280**, 20131865 (2013).

20. Martínez RN, Fernandez E, Alcober OA. A new non-mammaliaform eucynodont from the Carnian-Nornian Ischigualasto Formation, northwestern Argentina. *Rev Bras Paleontol* **16**, 61-76 (2013).

21. Martinelli AG, Eltink E, Da-Rosa AAS, Langer MC. A new cynodont from the Santa Maria formation, south Brazil, improves Late Triassic probainognathian diversity. *Papers in Palaeontology* **3**, 401-423 (2017).

22. Velazco PM, Buczek AJ, Novacek MJ. Two new tritylodontids (Synapsida, Cynodontia, Mammaliamorpha) from the Upper Jurassic, southwestern Mongolia. *Am Mus Novit* **3874**, 1-35 (2017).

23. Oliveira TV, Martinelli AG. New information about *Irajatherium hernandezi* Martinelli, Bonaparte, Schultz & Rubert 2005 (Eucynodontia, Trithelodontidae) from the Upper Triassic (Caturrita Formation, Paraná Basin) of Brazil. *Paläontologische Zeitschrift* **85**, 67-82 (2011).

24. Brocklehurst N. The Early Evolution of the Synapsida (Vertebrata, Amniota) and the Quality of Their Fossil Record. (ed^(eds). Humbolt-Universität zu Berlin (2015).

25. Sidor CA, Hancox PJ. *Elliotherium kersteni,* a new tritheledontid from the Lower Elliot Formation (Upper Triassic) of South Africa. *J Paleontol* **80**, 333-342 (2006).

26. Martinelli AG, Rougier GW. On *Chaliminia musteloides* (Eucynodontia: Trithelodontidae) from the Late Triassic of Argentina, and a phylogeny of Ictidosauria. *J Vert Paleontol* **27**, 442-460 (2007).

27. Fröbisch J. Global taxonomic diversity of anomodonts (Tetrapoda, Therapsida) and the terrestrial rock record across the Permian-Triassic boundary. *PLoS ONE* **3**, e3733 (2008).

28. Gao K-Q, Fox RC, Zhou C-F, Li D-Q. A new nonmammalian eucynodont (Synapsida: Therapsida) from the Triassic of northern Gansu Province, China, and its biostratigraphic and biogeographic implications. *Am Mus Novit* **3685**, 1-25 (2010).

29. Kammerer CF. A redescription of *Eriphostoma microdon* Broom, 1911 (Therapsida, Gorgonopsia) from the *Tapinocephalus* Assemblage Zone of South Africa and a review of Middle Permian gorgonopsians. In: *Early Evolutionary History of the Synapsida* (eds C.F. K, K.D. A, J. F). Springer (2014).

30. Kammerer CF. Systematics of the Rubidgeinae (Therapsida: Gorgonopsia). *PeerJ* **4**, e1608 (2016).

31. Kammerer CF. A new taxon of cynodont from the *Tropidostoma* Assemblage Zone (upper Permian) of South Africa, and the early evolution of Cynodontia. *Papers in Palaeontology* **2**, 387-397 (2016).

32. Kammerer CF. Anatomy and relationships of the South African gorgonopsian *Arctops* (Therapsida, Theriodontia. *Papers in Palaeontology* **3**, 583-611 (2017).

33. Castanhinha R*, et al.* Bringing dicynodonts back to life: paleobiology and anatomy of a new emydopoid genus from the Upper Permian of Mozambique. *PLoS ONE* **8**, e80974 (2013).

34. Kammerer CF, Fröbisch J, Angielczyk KD. On the validity and phylogenetic position of *Eubrachiosaurus browni,* a kannemeyeriiform dicynodont (Anomodontia) from Triassic North America. *PLoS ONE* **8**, e64203 (2013).

35. Brink KS, Reisz RR. Hidden dental diversity in the oldest terrestrial apex predator *Dimetrodon*. *Nat Commun* **5**, 3269 (2014).

36. Day MO. Middle Permian continental biodiversity changes as reflected in the Beaufort Group of South Africa: A bio- and lithostratigraphic review of the *Eodicynodon, Tapinocephalus* and *Pristerognathus* assemblage zones. (ed^(eds). University of the Witwatersrand (2014).

37. Liu J, Abdala F. Phylogeny and taxonomy of the Traversodontidae. In: *Early Evolutionary History of the Synapsida* (eds Kammerer CF, Angielczyk KD, Fröbisch J). Springer (2014).

38. Liu J, Abdala F. Therocephalian (Therapsida) and chroniosuchian (Reptilomorpha) from the Permo-Triassic transitional Guodikeng Formation of the Dalongkou Section, Jimsar, Xinjiang, China. *Vertebr Palasiat* **55**, 24-40 (2017).

39. Reisz RR, Frobisch J. The oldest caseid synapsid from the late Pennsylvanian of Kansas, and the evolution of herbivory in terrestrial vertebrates.(Report). *PLoS ONE* **9**, (2014).

40. Cox CB, Angielczyk KD. A new endothiodont dicynodont (Therapsida, Anomodontia) from the Permian Ruhuhu Formation (Songea Group) of Tanzania and its feeding system. *J Vert Paleontol* **35**, e935388 (2015).

41. Boos AD, Kammerer CF, Schultz CL, Soares MB, Ilha AL. A new dicynodont (Therapsida: Anomodontia) from the Permian of southern Brazil and its implications for bidentalian origins. *PLoS ONE* **11**, e0155000 (2016).

42. Kammerer CF, Bandyopadhyay S, Ray S. A new taxon of cistecephalid dicynodont from the upper Permian Kundaram Formation of India. *Papers in Palaeontology* **2**, 569-584 (2016).

43. Spindler F, Falconnet J, Fröbisch J. *Callibrachion* and *Datheosaurus*, two historical and previously mistaken basal caseasaurian synapsids from Europe. *Acta Palaeontol Pol* **61**, 597-616 (2016).

44. Viglietti PA, Smith RMH, Angielczyk KD, Kammerer CF, Fröbisch J, Rubidge BS. The *Daptocephalus* Assemblage Zone (Lopingian), South Africa: A proposed biostratigraphy based on a new compilation of stratigraphic ranges. *J Afr Earth Sci* **113**, 153-164 (2016).

45. Bordy E, Sciscio L, Abdala F, McPhee B, Choiniere J. First Lower Jurassic vertebrate burrow from southern Africa (upper Elliot Formation, Karoo Basin, South Africa). *Palaeogeogr, Palaeoclimatol, Palaeoecol* **468**, 362-372 (2017).

46. Gaetano LC, Abdala F, Govender R. The postcranial skeleton of the Lower Jurassic *Tritylodon longaevus* from southern Africa. *Ameghiniana* **54**, 1-36 (2017).

47. Kammerer CF, Smith RMH. An early geikiid dicynodont from the *Tropidostoma* Assemblage Zone (late Permian) of South Africa. *PeerJ* **5**, e2913 (2017).

48. Panciroli E, Walsh S, Fraser NC, Brusatte SL, Corfe I. A reassessment of the postcanine dentition and systematics of the tritylodontid *Stereognathus* (Cynodontia, Tritylodontidae, Mammaliamorpha), from the Middle Jurassic of the United Kingdom. *J Vert Paleontol* **37**, e1351448 (2017).

49. Day MO, Benson RB, Kammerer CF, Rubidge BS. Evolutionary rates of mid-Permian tetrapods from South Africa and the role of temporal resolution in turnover reconstruction. *Paleobiology* **44**, 347-367 (2018).

50. Sun A. Late Permian and Triassic terrestrial tetrapods of north China. *Vertebr Palasiat* **18**, 100-111 (1980).

51. Ponomarenko A*, et al.* Upper Jurassic Lagerstätte Shar Teg, southwestern Mongolia. *Paleontol J* **48**, 1573-1682 (2014).

52. Ottone EG*, et al.* A new Late Triassic age for the Puesto Viejo Group (San Rafael depocenter, Argentina): SHRIMP U–Pb zircon dating and biostratigraphic correlations across southern Gondwana. *J South Am Earth Sci* **56**, 186-199 (2014).

53. Sennikov A, Golubev V. Sequence of Permian tetrapod faunas of Eastern Europe and the Permian–Triassic ecological crisis. *Paleontol J* **51**, 600-611 (2017).

54. Mann A, Paterson RS. Cranial osteology and systematics of the enigmatic early 'sail-backed'synapsid Echinerpeton intermedium Reisz, 1972, and a review of the earliest 'pelycosaurs'. *J Syst Palaeontol*, 1-11 (2019).

55. Cohen KM, Finney SC, Gibbard PL, Fan J-X. The ICS international chronostratigraphic chart. *Episodes* **36**, 199-204 (2013).

56. Jones KE*, et al.* Fossils reveal the complex evolutionary history of the mammalian regionalized spine. *Science* **361**, 1249–1252 (2018).
