# Supplementary material for: Stepwise shifts underlie evolutionary trends in morphological complexity of the mammalian vertebral column
Source: Nat Commun. 2019 Nov 7;10:5071. doi: 10.1038/s41467-019-13026-3 (PMC6838112; doi:10.1038/s41467-019-13026-3)
Supplement: Supplementary file 2 — Reporting Summary [file 41467_2019_13026_MOESM2_ESM.pdf]

nature research

Corresponding author(s): Katrina Jones
Last updated by author(s): Sep 25, 2019

Reporting Summary

Nature Research wishes to improve the reproducibility of the work that we publish. This form provides structure for consistency and transparency in reporting. For further information on Nature Research policies, see Authors & Referees and the Editorial Policy Checklist.

Statistics

For all statistical analyses, confirm that the following items are present in the figure legend, table legend, main text, or Methods section.

n/a	Confirmed	
☐	☒	The exact sample size (n) for each experimental group/condition, given as a discrete number and unit of measurement
☐	☒	A statement on whether measurements were taken from distinct samples or whether the same sample was measured repeatedly
☐	☒	The statistical test(s) used AND whether they are one- or two-sided<br>*Only common tests should be described solely by name; describe more complex techniques in the Methods section.*
☒	☐	A description of all covariates tested
☐	☒	A description of any assumptions or corrections, such as tests of normality and adjustment for multiple comparisons
☐	☒	A full description of the statistical parameters including central tendency (e.g. means) or other basic estimates (e.g. regression coefficient) AND variation (e.g. standard deviation) or associated estimates of uncertainty (e.g. confidence intervals)
☐	☒	For null hypothesis testing, the test statistic (e.g. F, t, r) with confidence intervals, effect sizes, degrees of freedom and P value noted<br>*Give P values as exact values whenever suitable.*
☒	☐	For Bayesian analysis, information on the choice of priors and Markov chain Monte Carlo settings
☒	☐	For hierarchical and complex designs, identification of the appropriate level for tests and full reporting of outcomes
☒	☐	Estimates of effect sizes (e.g. Cohen's d, Pearson's r), indicating how they were calculated

Our web collection on statistics for biologists contains articles on many of the points above.

Software and code

Policy information about availability of computer code

Data collection	Raw data is from previously published study
Data analysis	R (3.4.2) with RStudio (v1.1.383) was used for all analyses. Packages are as follows:<br>Phylogeny: ape, phytools, paleotree,velociraptr, geomorph<br>Calculate complexity: regions<br>Analysis: mvMORPH, doParallel<br>Plotting: phytools, strap<br>Custom code was used for hypothesis testing and running on cluster. This code is available on github at https://github.com/katrinajones/<br>Functions

For manuscripts utilizing custom algorithms or software that are central to the research but not yet described in published literature, software must be made available to editors/reviewers. We strongly encourage code deposition in a community repository (e.g. GitHub). See the Nature Research guidelines for submitting code & software for further information.

Data

Policy information about availability of data

All manuscripts must include a data availability statement. This statement should provide the following information, where applicable:

- Accession codes, unique identifiers, or web links for publicly available datasets
- A list of figures that have associated raw data
- A description of any restrictions on data availability

Raw data are previously published and available on Dryad:
Jones KE, Angielczyk KD, Polly PD, Head JJ, Fernandez V, Lungmus JK, Tulga S, Pierce SE (2018) Data from: Fossils reveal the complex evolutionary history of the mammalian regionalized spine. Dryad Digital Repository. https://doi.org/10.5061/dryad.jm820mg
Additional data analyzed in this study, including complexity measures and phylogenies used, are available on Dryad at: https://doi.org/10.5061/dryad.5mkkwh71h

Field-specific reporting

Please select the one below that is the best fit for your research. If you are not sure, read the appropriate sections before making your selection.

☐ Life sciences ☐ Behavioural & social sciences ☒ Ecological, evolutionary & environmental sciences

For a reference copy of the document with all sections, see nature.com/documents/nr-reporting-summary-flat.pdf

Ecological, evolutionary & environmental sciences study design

All studies must disclose on these points even when the disclosure is negative.

Study description	Study examining the evolution of vertebral complexity in mammals.
Research sample	Sample includes vertebral measurements from 35 extant mammals, sampled to span the taxonomic diversity of the group, and 15 fossil non-mammalian synapsids. Fossils were selected based on preservation (complete vertebral columns), and to span the morphological transition from basal synapsids to mammals.
Sampling strategy	One specimen per species was used because the study focuses on cross-taxonomic levels, not within-taxon levels, of variation. Sampling was restricted due to the rarity of completely-preserved fossils, therefore our statistical analyses incorporated the influence of downsampling on hypothesis testing, in custom code described above.
Data collection	Linear and angular measures were taken directly from bones, or from digital models of bones in the software Mimics. Data collection was conducted by KJ.
Timing and spatial scale	NA
Data exclusions	No data were excluded
Reproducibility	Data is provided on Dryad, and code is available on Github.
Randomization	Monte Carlo simulation used for hypothesis testing
Blinding	NA

Did the study involve field work? ☐ Yes ☒ No

Reporting for specific materials, systems and methods

We require information from authors about some types of materials, experimental systems and methods used in many studies. Here, indicate whether each material, system or method listed is relevant to your study. If you are not sure if a list item applies to your research, read the appropriate section before selecting a response.

Materials & experimental systems

n/a	Involved in the study	
☒	☐	Antibodies
☒	☐	Eukaryotic cell lines
☐	☒	Palaeontology
☐	☒	Animals and other organisms
☒	☐	Human research participants
☒	☐	Clinical data

Methods

n/a	Involved in the study	
☒	☐	ChIP-seq
☒	☐	Flow cytometry
☒	☐	MRI-based neuroimaging

Palaeontology

Specimen provenance	No new specimens were collected as part of this study
Specimen deposition	Specimens are deposited in known Natural History collections, reported in Table S7.
Dating methods	No new dates were measured. However, first and last occurrence data were taken from the literature, as described in the methods and supplementary material, and used for time-calibrating the phylogeny in R. This data, along with the time-scaled phylogeny, will is deposited in Dryad.

☒ Tick this box to confirm that the raw and calibrated dates are available in the paper or in Supplementary Information.

Animals and other organisms

Policy information about studies involving animals; ARRIVE guidelines recommended for reporting animal research

Laboratory animals	None
Wild animals	None
Field-collected samples	Osteological collections in museums as described in the supplementary information
Ethics oversight	None required. Skeletons were already accessioned in collections prior to study.

Note that full information on the approval of the study protocol must also be provided in the manuscript.
